# Enlisting wild grass genes to combat nitrification in wheat farming: A nature-based solution

**DOI:** 10.1073/pnas.2106595118

**Published:** 2021-08-23

**Authors:** Guntur V. Subbarao, Masahiro Kishii, Adrian Bozal-Leorri, Ivan Ortiz-Monasterio, Xiang Gao, Maria Itria Ibba, Hannes Karwat, M. B. Gonzalez-Moro, Carmen Gonzalez-Murua, Tadashi Yoshihashi, Satoshi Tobita, Victor Kommerell, Hans-Joachim Braun, Masa Iwanaga

**Affiliations:** ^a^ Crop, Livestock and Environment Division, Japan International Research Center for Agricultural Sciences, Ibaraki 305-8686, Japan;; ^b^ Global Wheat Program, International Maize and Wheat Improvement Center, 56237 Texcoco, Mexico;; ^c^Department of Plant Biology and Ecology, University of the Basque Country, E-48080 Bilbao, Spain;; ^d^College of Bioresources Sciences, Nihon University, Kanagawa 252-0880, Japan

**Keywords:** BNI, nitrogen pollution, nitrification inhibition, genetic improvement, wheat

## Abstract

Active nitrifiers and rapid nitrification are major contributing factors to nitrogen losses in global wheat production. Suppressing nitrifier activity is an effective strategy to limit *N* losses from agriculture. Production and release of nitrification inhibitors from plant roots is termed “biological nitrification inhibition” (BNI). Here, we report the discovery of a chromosome region that controls BNI production in “wheat grass” *Leymus racemosus* (Lam.) Tzvelev, located on the short arm of the “Lr#3Ns^b^” (Lr#n), which can be transferred to wheat as T3BL.3Ns^b^S (denoted Lr#n-SA), where 3BS arm of chromosome 3B of wheat was replaced by 3Ns^b^S of *L. racemosus*. We successfully introduced T3BL.3Ns^b^S into the wheat cultivar “Chinese Spring” (CS-Lr#n-SA, referred to as “BNI-CS”), which resulted in the doubling of its BNI capacity. T3BL.3Ns^b^S from BNI-CS was then transferred to several elite high-yielding hexaploid wheat cultivars, leading to near doubling of BNI production in “BNI-MUNAL” and “BNI-ROELFS.” Laboratory incubation studies with root-zone soil from field-grown BNI-MUNAL confirmed BNI trait expression, evident from suppression of soil nitrifier activity, reduced nitrification potential, and N_2_O emissions. Changes in *N* metabolism included reductions in both leaf nitrate, nitrate reductase activity, and enhanced glutamine synthetase activity, indicating a shift toward ammonium nutrition. Nitrogen uptake from soil organic matter mineralization improved under low N conditions. Biomass production, grain yields, and *N* uptake were significantly higher in BNI-MUNAL across *N* treatments. Grain protein levels and breadmaking attributes were not negatively impacted. Wide use of BNI functions in wheat breeding may combat nitrification in high *N* input–intensive farming but also can improve adaptation to low *N* input marginal areas.

## Significance

Globally, wheat farming is a major source of nitrogen pollution. Rapid generation of soil nitrates cause nitrogen leakage and damage ecosystems and human health. Here, we show the 3Ns^b^S chromosome arm in wild grass *(Leymus racemosus)* that controls root nitrification inhibitor production can be transferred into elite wheat cultivars, without disrupting the elite agronomic features. Biological nitrification inhibition (BNI)–enabled wheats can improve soil ammonium levels by slowing down its oxidation and generate significant synergistic benefits from assimilating dual nitrogen forms and improving adaptation to low N systems. Deploying BNI-enabled wheat on a significant proportion of current global wheat area (ca. 225 M ha) could be a powerful nature-based solution for reducing N fertilizer use and nitrogen losses while maintaining productivity.

Nitrification and denitrification are critical soil biological processes, which, left unchecked, can accelerate generation of harmful reactive nitrogen (N) forms (NO_3_^−^, N_2_O, and NOx) that trigger a “nitrogen cascade,” damaging ecosystems, water systems, and soil fertility (1–8). Excessive nitrifier activity and a rapid generation of soil nitrates plague modern cereal production systems. This has led to shifting crop *N* nutrition toward an “all nitrate form,” which is largely responsible for *N* losses and a decline in agronomic nitrogen-use efficiency (NUE) (6, 7, 9–11).

Wheat, one of the three founding crops for food security (12), consumes nearly a fifth of factory-produced *N* fertilizers, and it has an average NUE of 33%, which has remained unchanged for the last two decades (13–15). Regulating soil nitrifier activity to slow the rate of soil nitrate formation should provide more balanced *N* forms (NH_4_^+^ and NO_3_^−^) for plant uptake (rather than nearly “all NO_3_^−^” at present), reduce *N* losses, and facilitate the assimilation of dual *N* forms. This optimizes the utilization of biochemical machinery for *N* assimilation, improving stability and possibly enhancing yield potential (16). In addition, the assimilation of NH_4_^+^ is energetically more efficient (requiring 40% less metabolic energy) than NO_3_^−^ assimilation (16). Often, a stimulatory growth response is observed in wheat, when 15 to 30% of NO_3_^−^ is replaced with NH_4_^+^ in nutrient solutions (17, 18).

Synthetic nitrification inhibitors (SNIs) have been shown to suppress N_2_O emissions, reduce *N* losses, and improve agronomic NUE in several cereal crops including wheat (6, 19–21). However, the lack of cost effectiveness, inconsistency in field performance, inability to function in tropical environments, and the concerns related to the entering of SNIs into food chains have limited their adoption in production agriculture (6, 7, 19, 20).

Biological nitrification inhibition (BNI) is a plant function whereby nitrification inhibitors (BNIs) are produced from root systems to suppress soil nitrifier activity (22–26). Earlier, we reported that the BNI capacity in the root systems of cultivated wheat lack adequate strength to effectively suppress soil nitrifier activity in the rhizosphere (24, 25). *Leymus racemosus* (hereafter referred to as “wild grass”), a perennial Triticeae evolutionarily related to wheat, produces extensive root systems ( *SI Appendix*, Fig. S1) and was discovered to have a high BNI capacity several times higher than cultivated wheat. It was also effective in suppressing soil nitrifier activity and in reducing soi -nitrate formation ( *SI Appendix*, Fig. S2) (25). Subsequently, the chromosome Lr#n = 3Ns^b^ was found to be controlling a major part of BNI capacity in wild grass, and it is the focus of our current research (25, 27, 28). Earlier, we reported that Lr#I and Lr#J had a minor impact on BNI capacity, but they are not the focus of this research (25).

We transferred the Lr#n chromosome (Lr#n-SA = T3BL.3Ns^b^S) controlling BNI capacity (hereafter referred to as BNI trait) into the cultivated wheat, Chinese Spring (CS). The results of the transfer of this BNI trait into several elite wheat types with a grain-yield (GY) potential >10 t ha^−1^, resulting in substantial improvements of BNI capacity in root systems, are reported in this paper.

## Results

### BNI Capacity Has Not Increased over Five Decades of Wheat Breeding.

We evaluated 20 International Maize and Wheat Improvement Center (CIMMYT)–derived wheat varieties released between 1950 and 2010 (belonging to both pre-Green Revolution [GR] and post-GR era wheat varieties) to determine the impact of five decades of breeding under high nitrogen input conditions on the BNI capacity of wheat root systems ( *SI Appendix*, Study 1). We observed no clear trend in the 20 varieties’ BNI capacity ( *SI Appendix*, Fig. S3). There were significant differences (*P <* 0.001) in the BNI capacities of wheat varieties ( *SI Appendix*, Table S1), but none showed higher BNI capacity than the standard wheat genetic stock, CS, an old landrace from China ( *SI Appendix*, Table S1). Some elite varieties released in 2000 (such as ROELFS) as well as “SONORA-64” (an early GR era variety released in 1964) have BNI capacity akin to CS (ranging from 70 to 90 allylthiourea unit [ATU] ⋅ g^−1^ root dryweight d^−1^). Also, these results indicate that wheat breeding had no directional impact (i.e., neither positive nor negative) on the BNI capacity of root systems (i.e., either selected “for” or “against”) ( *SI Appendix*, Fig. S3). This, in turn, could be due to a lack of adequate allelic variation for this trait (i.e., BNI strength necessary to suppress soil nitrifier activity) in wheat or close wheat relatives used in breeding.

### Genetic Sources for BNI Capacity Identified in Perennial Wild Grasses.

**Fig. 1.**
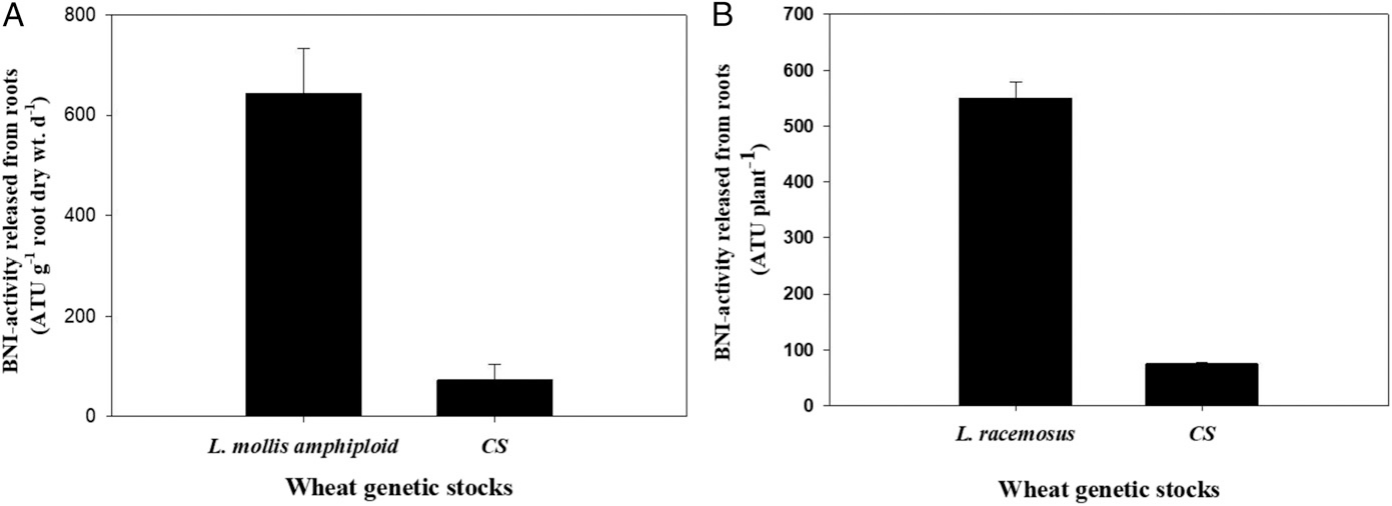
BNI activity in *L. mollis* and *L. racemosus* compared to CS. (*A*) Nearly sevenfold higher BNI activity is released from *L. mollis* amphiploid (*L. mollis × T. turgidum*) compared to cultivated wheat cv. CS. Values are means ± SE of four replications. Plants were grown hydroponically for 60 d in a walk-in growth chamber before collecting root exudates to determine BNI release ( *SI Appendix*, Study 2b). (*B*) About eightfold higher BNI activity is released from roots of wild grass (*L. racemosus*) compared to cultivated wheat cv. CS. For *L. racemosus*, plants were grown in pots filled with soil (Andosol; 13 kg soil pot^−1^) for 1 y before using them for root exudate collection (see *SI Appendix*, Fig. S1 for pictures of root systems of wild grass and field-grown cultivated wheat, MUNAL root systems). For cultivated wheat (CS), plants were grown hydroponically for 60 d in a walk-in growth chamber as described in *Materials and Methods*, and root exudates were collected from intact plant root systems to determine BNI activity released (data from results of experiment 5a). Values are means ± SE of four replications ( *SI Appendix*, Study 2a).

Of 20 amphiploids (generated by crossing various wild wheat species and wild grasses with cultivated wheat) evaluated for BNI capacity ( *SI Appendix*, Study 2a and b), only the *Leymus mollis* (Trin.) Pilger (4× = 28, genomes NsNsXmXm) amphiploid (generated by crossing with *Triticum turgidum*) showed BNI capacity several times (around seven times) higher than CS (Fig. 1*A* and *SI Appendix*, Table S2 *A–C* ). This is akin to what was reported earlier for another wild grass species *L. racemosus* (Lam.) Tzvelev (4× = 28, genomes NsNsXmXm) and from the results reported here (Fig. 1*B*). Both perennial wild grass relatives are part of the tertiary gene pool of wheat (i.e., distant relatives of wheat), posing major practical challenges for the transfer of BNI genes to wheat. No other wild relatives tested in this study showed higher BNI capacity than CS ( *SI Appendix*, Table S2*C* ). Negative BNI activity was detected in the root exudates of most amphiploids derived from wild wheats, implying that they are likely to stimulate nitrifying bacteria, thus accelerating soil nitrification ( *SI Appendix*, Table S2*C* ). So far, only two wild grasses (*L. racemosus* and *L. mollis*) have been identified as genetic sources for improving the BNI capacity in the root systems of cultivated wheat.

### Identification of Chromosomal Region Controlling BNI Capacity in *L. racemosus*.

**Fig. 2.**
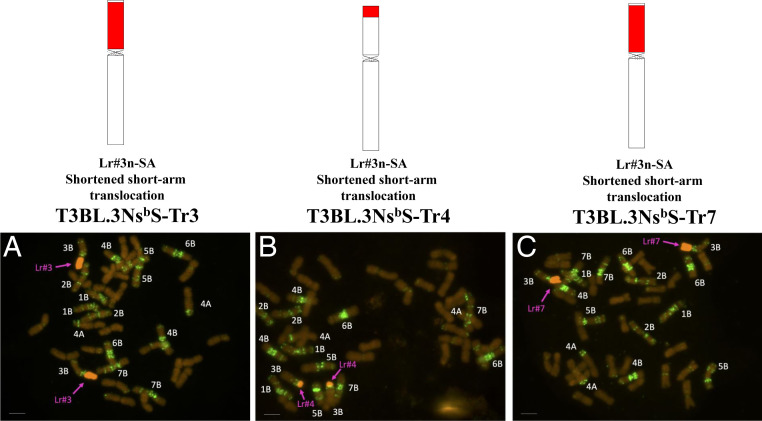
Lr#n short-arm recombinants with shorter fragment size than Lr#n-SA in CS. Chromosome analysis of three recombinants. (*A*) Lr-n-SA-Tr3 (red: *L. racemosus* genomic DNA, green: mixture of AAG and Tail probes). (*B*) Lr-n-SA-Tr4 (red: *L. racemosus* genomic DNA, green: mixture of AAG and Tail probes). (*C*) Lr-n-SA-Tr7 (red: *L. racemosus* genomic DNA, green: mixture of AAG and Tail probes). (Scale bar, 10 μm.)

We reported earlier that the high BNI capacity of *L. racemosus* was controlled by chromosome Lr#n (25). The BNI capacity is conferred by the short arm of Lr#n; this was evident from the doubling (*P* < 0.001) of BNI capacity when the short arm was introduced into CS but not with long-arm translocation (Table 1 and *SI Appendix*, Study 4a and Fig. S4 *A* and *B* ). A previous study established that the short arm of Lr#n corresponds to a *Leymus* chromosome from homoeologous group 3 (3Ns) (28). In addition, we tested two independent translocations of the complete 3Ns^b^S arm to chromosomes 7B and 3B to determine which translocation position maximizes BNI trait expression ( *SI Appendix*, Fig. S4*C* *)*. The BNI trait from Lr#n fully expressed (*P < 0.001*) only on wheat 3B translocation (T3BL.3Ns^b^S), not on 7B translocation (T7BL.3Ns^b^S) (Table 1). Three 3Ns^b^S recombinant chromosomes with reduced 3Ns^b^ arm sizes (T3BL.3Ns^b^S-Tr-3; T3BL.3Ns^b^S-Tr-4; T3BL.3Ns^b^S-Tr-7) were developed in CS using the *ph1b* mutation (Fig. 2 and *SI Appendix*, Study 4b and Fig. S5). BNI activity release rates in two recombinant chromosomes were 20 to 40% higher (*P* < 0.001) compared to T3BL.3Ns^b^S (Table 2), suggesting that by reducing the size of the 3Ns^b^S arm, BNI trait expression can be further improved by minimizing negative impacts from other wild genes accompanying the BNI trait.

**Table 1.** BNI capacity of wheat *Leymus* genetic stocks ( *SI Appendix*, Study 3a)

Serial no.	Genetic stock details	BNI activity released from intact plant roots (ATU ⋅ g^−1^ root dry wt. ⋅ d^−1^)	
		Mean	SE
1	CS	77.7^a^	2.9
2	Lr#n-addition (CS*2/LE.RA)	162.9^b^	17.7
3	Lr#n-Short-arm Tr.-7B	80.0^a^	4.2
4	Lr#n-Short-arm Tr.-3B (T3BL.3Ns^b^S)	168.6^b^	7.4
5	Lr#n-Long-Arm Tr. (CS*2/LE.RA//2*WBLL1/3/CS)	79.3^a^	3.7
	SEM (*P* < 0.05) (based on two-way analysis General Linear Model using SYSTAT 14.0)	9.34 (*P* < 0.05)	

Holm–Sidak method—letters represent values that are significantly different (*P* < 0.05). Values are means ± SE of four replications.

**Table 2.** BNI capacity of Lr#n short-arm translocations (with shortened short arm of chromosome Lr#n-SA) in CS genetic background along with CS ( *SI Appendix*, Study 4b)

Serial no.	Genetic stock details	Genetic stock explanation	BNI activity released from intact plant roots (ATU ⋅ g^−1^ root dry wt. ⋅ d^−1^)		Percent of CS control
			Mean	SE	
1	CS		57.4^a^	3.8	100
2	CSMONO3B//CS*2/LE.RA Lr#n-SA) (T3BL.3Ns^b^S)	N complete short arm	113.0^b^	4.5	196
3	CSMONO3B//CS*2/LE. RA Shortened short-arm translocation-3 (T3BL.3Ns^b^S-Tr3)	N shortened SA Tr-3	137.4^c^	2.6	239
4	CSMONO3B//CS*2/LE.RA/3/CS ph ph 1b Shortened short-arm translocation-4 (T3BL.3Ns^b^S-Tr4)	N shortened SA Tr-4	97.6^b^	9.3	170
5	CSMONO3B//CS*2/LE.RA/3/CS ph ph 1b Shortened short-arm translocation-7 (T3BL.3Ns^b^S-Tr7)	N shortened SA Tr-7	163.9^c^	5.9	286
	SE of Least Square Mean (*P* < 0.001) (based on two-way analysis General Linear Model using SYSTAT 14.0)	6.86 (*P* < 0.001)			

Holm–Sidak method—letters represent values that are significantly different (*P* < 0.001). Values are means ± SE of four replications.

### Transfer of T3BL.3Ns^b^S into Elite Wheats Conferring BNI Capacity.

**Fig. 3.**
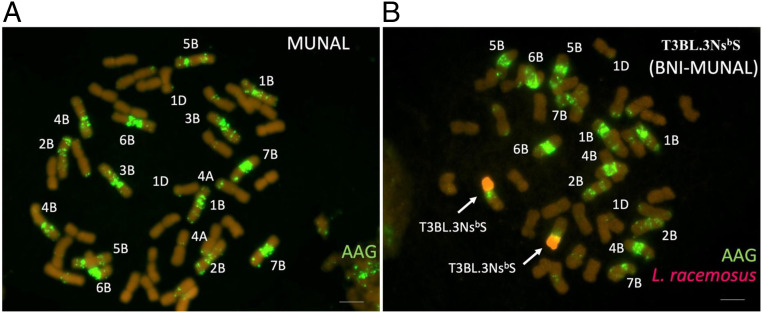
Karyotype analysis of BNI isogenic wheat lines. (*A*) Wheat line MUNAL. (*B*) BNI-MUNAL carrying Lr#n-SA translocation (complete short arm) on wheat chromosome 3B (T3BL.3Ns^b^S) Genomic in situ hybridization (GISH)/Florescence in situ hybridization (FISH) (red: *L. racemosus* genomic DNA, green AAG probe).

**Fig. 4.**
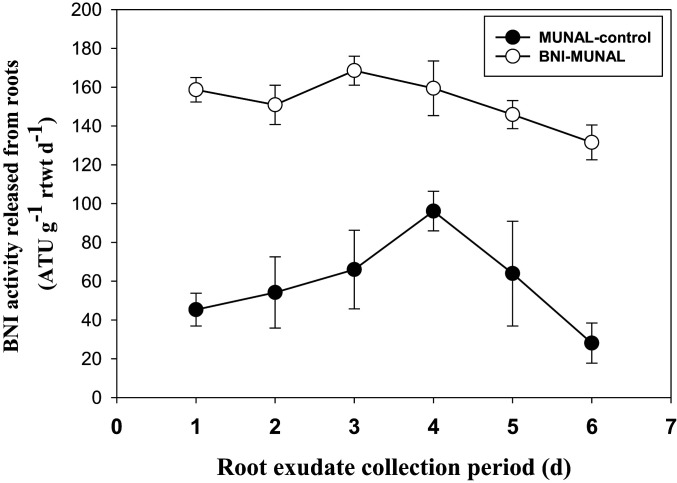
Two- to fivefold higher BNI activity is released from BNI-MUNAL (i.e., MUNAL carrying T3BL.3Ns^b^S) compared to MUNAL control (BNI isogenic lines) during a 6-d monitoring period using various root exudate trap solutions ( *SI Appendix*, Study 5b). 1) RE-NH_4_-1 (1.8 L aerated solutions of 0.5 mM NH_4_Cl + 200 μM CaCl_2_ for 24 h—first day collection); 2) RE-nutr-NH_4_-1 (1.8 L aerated solutions of one-quarter strength nutrient solution with 0.5 mM NH_4_Cl for 24 h—second day collection); 3) RE-water-1 (1.8 L aerated solutions of 200 μM CaCl_2_ for 24 h—third day collection); 4) RE-NH_4_-2 (1.8 L aerated solutions of 1.0 mM NH_4_Cl + 200 μM CaCl_2_ for 24 h—fourth day collection); 5) RE-nutr-NH_4_-2 (1.8 L aerated solutions of one-quarter strength nutrient solution with 1.0 mM NH_4_Cl for 24 h—fifth day collection); and 6) RE-water-2 (1.8 L aerated solutions of 200 μM CaCl_2_ for 24 h—sixth day collection). Values are means ± SE from four replications.

The wheat *L.racemosus* T3BL.3Ns^b^S chromosome was successfully transferred from CS into several elite hexaploid wheat cultivars: “ROELFS,” “MUNAL,” “NAVOJOA,” and “QUAIU,” with a GY potential of >10 t ⋅ ha^−1^ ( *SI Appendix*, Tables S2*D* and S3 and https://www.orderseed.cimmyt.org/iwin/iwin-results-1.php). This was achieved by utilizing at least four backcrosses and selection for T3BL.3Ns^b^S using fluorescence in situ hybridization following Kishii et al. (29) (Fig. 3 *A* and *B* and *SI Appendix*, Fig. S6 *A* and *B* ). We conformed an enhanced BNI capacity, as there were significant improvements (*P* < 0.001) in BNI activity release from the root systems of the most BNI elite wheats (Table 3 and *SI Appendix*, Study 5a). We also observed a near doubling (*P* < 0.001) in the release of BNI activity from root systems of BNI-MUNAL and BNI-ROELFS (compared to MUNAL control and ROELFS control). For “BNI-QUAIU,” we observed only a 50% increase (*P* < 0.001) in BNI activity release. For “BNI-NAVOJOA,” there was no significant improvement in BNI release compared to “NAVOJOA control” (Table 3), indicating that BNI trait expression is wheat genetic background dependent. Subsequent studies ( *SI Appendix*, Study 5b) with BNI-MUNAL revealed that BNI release rates were between two and five times higher than in MUNAL control (monitored over 6 d, during which time root exudates were collected using different trap solutions), indicating enormous plasticity in the phenotypic expression of BNI trait (Fig. 4). Such plasticity in the magnitude of BNI release is needed to deliver the required dosage of BNIs (determined by amounts of NH_4_^+^ available at soil sites) for suppressing and/or moderating nitrifier activity (23, 26); root systems constantly face the challenge of temporal and spatial variation in rhizosphere environment.

**Table 3.** BNI capacity of elite wheat genetic stocks and BNI elite wheats ( *SI Appendix*, Study 5a)

Serial no.	Wheat genetic stock	BNI activity released from intact plant roots (ATU ⋅ g^−1^ root dry wt. ⋅ d^−1^)	
		Mean	SE
1	ROELFS	86.5^a^	12.8
2	BNI-ROELFS (CSMONO3B/3/CS/LE.RA/CS/4/CSph/5/5*ROELFS(N)	162.2^b^	16.8
	SE of Least Square Mean (*P* < 0.001) (based on two-way analysis General Linear Model using SYSTAT 14.0)	6.99 (P < 0.005)	
3	MUNAL	92.7^a^	12.1
4	BNI-MUNAL (CSMONO3B/3/CS/LE.RA/CS/4/CS/ph/5/5*MUNAL(N)	181.7^b^	22.3
	SE of Least Square Mean (*P* < 0.001) (based on two-way analysis General Linear Model using SYSTAT 14.0)	17.9 (*P* < 0.05)	
5	NAVOJOA	91.2^a^	22.4
6	BNI-NAVOJOA(CSMONO3B/3/CS/LE.RA/CS/4/CS/ph/4/4*NAVAJOA(N)	119.2^a^	14.2
	SE of Least Square Mean (*P* < 0.001) (based on two-way analysis General Linear Model using SYSTAT 14.0)	22.42^ns^	
7	QUAIU	70.2^a^	4.9
8	BNI-QUAIU CSMONO3B/3/CS/LE.RA/CS/4/CSph/5/5*Quaiu(N)	126.4^b^	11.8
	SE of Least Square Mean (*P* < 0.001) (based on two-way analysis General Linear Model using SYSTAT 14.0)	11.19 (*P* < 0.05)	

Holm–Sidak method—letters represent values that are significantly different (*P* < 0.05). Values are means ± SE of four replications.

### BNI Trait (T3BL.3Ns^b^S) Suppresses Nitrification and Improves *N* Uptake, Biomass Production, and GY in a Range of Nitrogen Inputs under Field Conditions—Proof of Concept.

Based on conservative estimates of root biomass being 1.95 Mg ⋅ ha^−1^ (assuming that 10% of the total aboveground biomass measured is allocated to roots) with maximum BNI activity release rates of 182 ATU ⋅ g^−1^ root dry weight d^−1^ (Table 3 and Fig. 4), we estimate that 354.9 × 10^6^ ATU ⋅ ha^−1^ ⋅ d^−1^ can potentially be released from the root systems of BNI-MUNAL at its peak (i.e., booting stage—GS51, Zadoks scale), measured in hydroponics (30). This estimate amounts to an inhibitory potential equivalent to the application of 212.9 g nitrapyrin ha^−1^ ⋅ d^−1^ [based on 1 ATU being equivalent to 0.6 μg of nitrapyrin (26, 31)]. Such high levels of BNI release may not be sustained over extended periods under field conditions. Nevertheless, this is large enough to have significant suppressive effect on nitrifier populations.

**Fig. 5.**
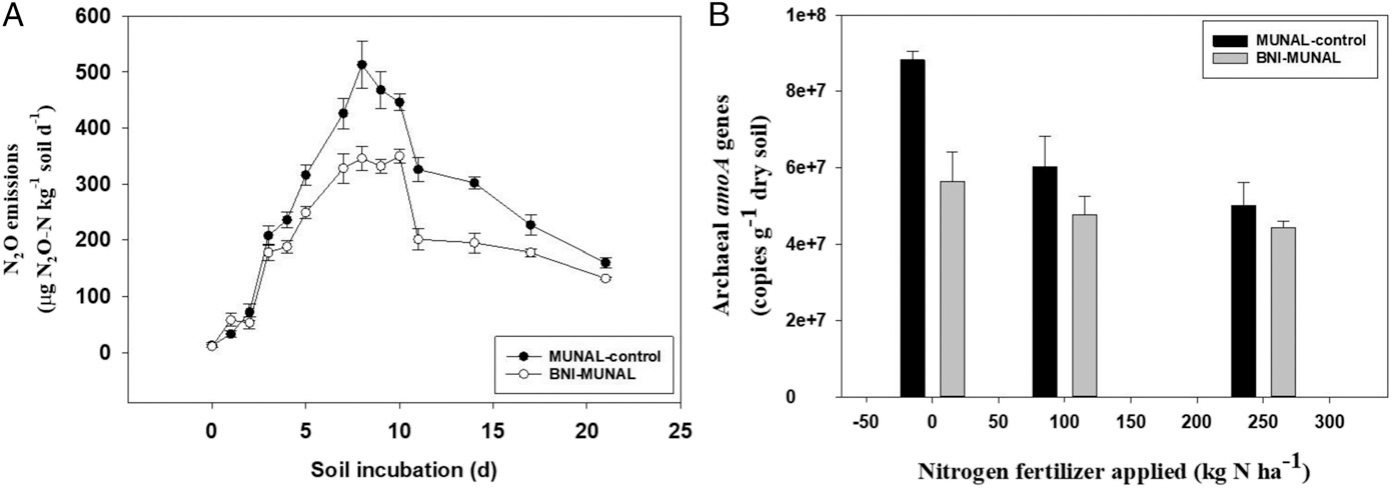
BNI function impact on N_2_O emissions and nitrifying populations in root-zone soils of field-grown wheat lines ( *SI Appendix*, Study 6a). (*A*) N_2_O emissions from root-zone soils of BNI isogenic lines, MUNAL control versus BNI-MUNAL. The root-zone soils used in this study were collected from MUNAL control and BNI-MUNAL (from 250 kg ⋅ N ⋅ ha^−1^ field plots). A total of 5 g air-dried soil was incubated with 250 ppm N [as (NH_4_)_2_SO_4_] using a 100-mL glass vial at 20 °C with 80% relative humidity in the incubator; soil moisture levels were maintained at 60% water-filled pore space during the incubation period. Values are means ± SE of four replications (see *SI Appendix*, Fig. S7*B* for cumulative N_2_O emissions over the 21-d period and for a statistical analysis of these results). (*B*) Influence of BNI-MUNAL on AOA populations in root-zone soils of field-grown plants. These results suggest that BNI-MUNAL suppressed AOA ranging from 20 to 36% depending on the nitrogen treatment of field plots. Root-zone soil samples were taken 16 d after the application of the second split nitrogen fertilizer. Values are means ± SE of four replications. Based on a three-way analysis of data using a General Linear Model model with SYSTAT 14.0; significant (*P* < 0.005) genetic stock effect on AOA; significant (*P* < 0.005) nitrogen treatment effect on AOA populations in rhizosphere soils.

Field studies on acidic soils (soil pH 5.0 to 5.5) at Japan International Research Center for Agricultural Sciences (JIRCAS; Tsukuba, Japan; *SI Appendix*, Study 6a) indicated a 30% reduction in soil nitrate levels (*P* < 0.05) and substantial improvements in soil ammonium levels (*P* < 0.001) (in core soil samples taken at a 20-cm depth near plant roots) ( *SI Appendix*, Table S4*A* ) (compared to MUNAL control field plots), indicating the expression of BNI function in BNI-MUNAL root systems. In root-zone soils (defined as “soil that is in close proximity to roots”) ( *SI Appendix*, Fig. S7*A*, 1), the nitrate percentage of inorganic N pool (%) declined by 26% (*P* < 0.001), potential net nitrification rates declined by 17% (*P* < 0.05) (Table 4), and potential nitrification declined by 28% ( *SI Appendix*, Fig. S7*A* ). The slopes of regression lines are significantly different (*P* < 0.001) based on analysis of covariance (ANCOVA). Also, N_2_O emissions based on laboratory incubation studies declined by 25% (*P* < 0.01) (Fig. 5*A*; *SI Appendix*, Fig. S7*B* ) and soil archaea (AOA) populations declined by 20 to 36% (*P* < 0.005) (Fig. 5*B*). BNI function had a stronger inhibitory effect on archaea compared to ammonium oxidizing bacteria (AOB) populations, as AOBs did not show significant decline in BNI-MUNAL ( *SI Appendix*, Fig. S7*C* ). This lends support to recent reports of BNIs being more potent on AOAs (32, 33), whereas SNIs are more effective on AOBs (34). Furthermore, soil microcosm studies ( *SI Appendix*, Study 6b) with alkaline soils suggested a 45% decline (*P* < 0.05) in AOBs with BNI-MUNAL but did not influence AOAs ( *SI Appendix*, Table S4*B* *).*

**Table 4.** Nitrate percentage of inorganic N pool and potential net nitrification rates in root-zone soils of field-grown plants of BNI isogenic lines, MUNAL control versus BNI-MUNAL after 21-d incubation period ( *SI Appendix*, Study 6a)

Wheat genetic stocks	Soil ammonium (μg ⋅ g^−1^ soil)		Soil nitrate (μg ⋅ g^−1^ soil)		Total inorganic N (NH_4_^+^ + NO_3_^−^) μg ⋅ g^−1^ soil		Nitrate percentage of inorganic N pool (%)		Potential net nitrification rate (μg ⋅ NO_3_^−^ ⋅ g^−1^ soil ⋅ d^−1^)	
	Mean	SE	Mean	SE	Mean	SE	Mean	SE	Mean	SE
MUNAL control	86.4	3.2	505.0	31.1	591.4	28.2	85.4	1.2	24.05	0.74
BNI-MUNAL	245.0	23.0	422.5	22.2	667.5	15.0	63.3	3.2	20.10	0.52
SE of LS mean (based on two-way analysis GLM using SYSTAT 14.0)	8.9**		18.1*				1.44**		0.87*	

The nitrate percentage of inorganic N pool is calculated as [soil − NO_3_^−^/(soil NO_3_^−^+ soil NH_4_^+^)] × 100], that is, proportion of nitrate to total inorganic N. Initial soil inorganic nitrogen levels (NO_3_^−^ and NH_4_^+^) are deducted before calculating net change in soil inorganic *N* forms during the incubation period. Potential net nitrification rate = [soil nitrate levels at the end of incubation period (i.e., at 21 d) – soil nitrate levels at the beginning of incubation period (i.e., time “0”)]/21. Based on Holm–Sidak test; **significant at *P* < 0.001*;* *significant at *P* < 0.05. Values are means ± SE of four replications.

However, this requires additional studies because most available evidence indicates that BNI function is mostly effective in soils that are acidic or neutral (5, 6, 23–26). Functionally, AOAs are most active and dominant in acid soils (34), whereas AOBs are active and dominate in neutral alkaline soils (35–37). The possibility for BNI trait expression under a wide range of soil pH conditions can potentially expand the scope for soil types in which BNI wheats can be deployed. The above observations were, however, based on laboratory incubation studies using root-zone soils from field-grown plants. The magnitude of BNI impact on bulk soils remains unknown, as is the BNI pathway that could influence nitrifier populations beyond the rhizosphere root zone.

**Fig. 6.**
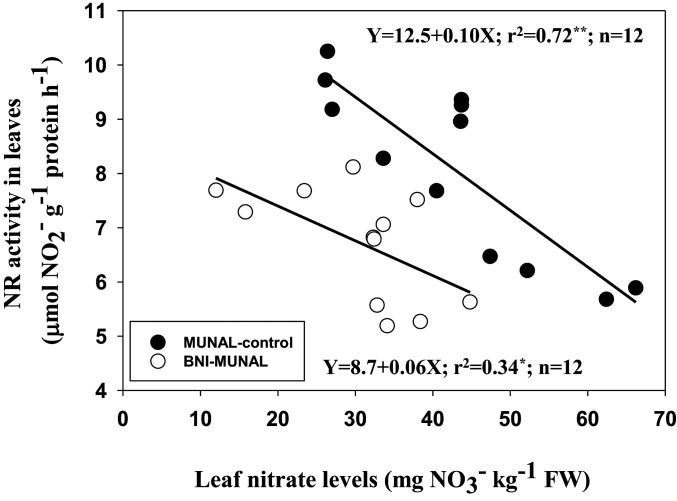
Relationship between leaf nitrate levels and NRA activity in field-grown BNI isogenic lines, MUNAL control, and BNI-MUNAL ( *SI Appendix*, Study 6a). The slopes of regression lines are significantly different (*P* < 0.001) based on ANCOVA. Leaf sample data from all three nitrogen treatments are used in this presentation; first sampling data are used. NRA and leaf nitrate analysis from leaf samples collected from four plants for each experimental plot represent each data point. Leaf nitrate levels and NRA levels were substantially lower in BNI-MUNAL compared to MUNAL control. The relationship between NRA and leaf nitrate levels is fundamentally different in BNI-MUNAL compared to MUNAL control. Also, see *SI Appendix*, Tables S5 and S6 for detailed results on NRA and leaf nitrate levels and for the statistical analysis of results.

In addition, nitrogen metabolism in BNI-MUNAL was fundamentally altered. This is evident from radical changes in the relationship between leaf nitrate levels and nitrate reductase activity (NRA) in BNI-MUNAL (compared to MUNAL control) ( *SI Appendix*, Study 6a). The slopes of regression lines are significantly different (*P* < 0.001) based on ANCOVA (Fig. 6). Further, BNI trait introduction led to a substantial decline in leaf nitrate levels (about 30 to 40%; *P* < 0.001; *SI Appendix*, Table S5) and leaf NRA (around 20%; *P* < 0.001) ( *SI Appendix*, Table S6) and an increase (about 15%; *P* < 0.001) in glutamine synthetase activity (GSA) in leaves; GSA is an enzyme that is at the forefront of ammonium assimilation ( *SI Appendix*, Table S6). Likely due to enhanced ammonium nutrition (uptake and assimilation), the root-zone soil pH was consistently lower (about 0.1 to 0.2 unit; *P* < 0.001) in BNI-MUNAL ( *SI Appendix*, Fig. S7*D* ).

**Fig. 7.**
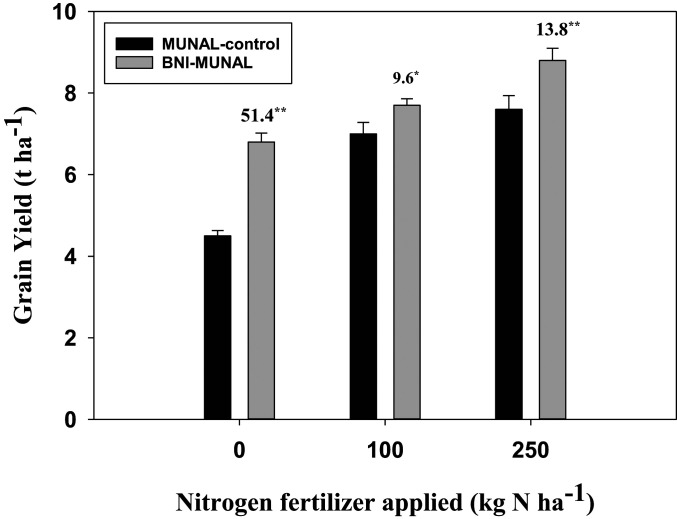
GY of BNI isogenic lines, MUNAL control, and BNI-MUNAL under various nitrogen fertilizer applications in the field ( *SI Appendix*, Study 6a). Three-way ANOVA using General Linear Model with SYSTAT 14.0; SE of Least Square mean (genetic stock) 0.164 (*P* < 0.001); SE of LS mean (*N*-Tr) 0.201 (*P* < 0.001); values are means ± SE of four replications. **P < 0.001; *P < 0.05.

BNI-MUNAL had improved total biomass production (*P* < 0.001) and GY (*P* < 0.001) across treatments (Fig. 7 and *SI Appendix*, Study 6a and Table S7) based on field evaluations at the JIRCAS experimental station in 2019, with improved (*P* < 0.001) agronomic attributes: harvest index, tiller numbers, and 100 seed wt. ( *SI Appendix*, Table S7 *)*. The biggest impact from introducing a BNI trait is evident in no N application field plots in which *N* deficiency symptoms are visible (also based on Soil Plant Analysis Development chlorophyll meter readings that reflect chlorophyll and nitrogen content in leaves) only in MUNAL control but not in BNI-MUNAL ( *SI Appendix*, Fig. S8 *A*–*C* *)*. Its biomass production and GYs were 50% higher (*P* < 0.001) than MUNAL control (Fig. 7 and *SI Appendix*, Table S7 *;* *SI Appendix*, Fig. S8*D* ). With *N* fertilization (100 to 250 kg ⋅ N ⋅ ha^−1^), BNI-MUNAL yielded about 10 to 14% (*P* < 0.001) higher than MUNAL control (Fig. 7). This is possibly due to improved NH_4_^+^ assimilation, which is energetically more efficient than NO_3_^−^ assimilation (16) and can have a synergistic impact on growth and GYs. Supplemental NH_4_^+^ in nutrient solutions has been reported to stimulate growth in wheat and maize (15, 17).

**Fig. 8.**
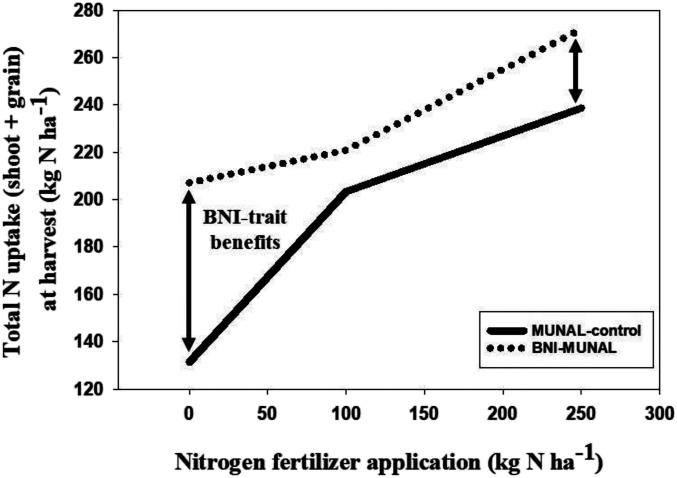
Total nitrogen uptake (based on aboveground dry matter that includes grain) in wheat BNI isogenic lines (MUNAL control and BNI-MUNAL) under various nitrogen fertilizer applications in the field ( *SI Appendix*, Study 6a). Introduction of BNI trait (T3BL.3Ns^b^S) resulted in substantial improvements in nitrogen uptake in BNI-MUNAL compared to MUNAL control. Three-way ANOVA using General Linear Model with SYSTAT 14.0; SE of Least Square mean (genetic stock) 5.79 (*P <* 0.001); SE of LS of mean (*N*-Tr) 7.10 (*P* < 0.001)—see *SI Appendix*, Fig. S9*B* for detailed results on total N uptake with a statistical analysis of results and their significance). Values are means ± SE of four replications.

Nitrogen uptake (aboveground biomass that includes grain) improved by about 28% (*P* < 0.001) (ranging from 9 to 58% depending on *N* fertilizer treatment) in BNI-MUNAL (Fig. 8 and *SI Appendix*, Study 6a and Fig. S9 *A* and *B* ). It is likely that the root systems of perennial wild grass have the ability to mineralize N more efficiently from soil organic matter (SOM) than cultivated wheat, as they are highly adapted to low fertility and low N environments (38). Efficient *N* uptake from SOM can be part of the adaptation to low N environments. The exceptional performance of BNI-MUNAL under low N conditions (58% higher N uptake; *P* < 0.001) supports the hypothesis that T3BL.3Ns^b^S, in addition to the BNI trait, is also carrying genes that improve the uptake of native soil N by efficient SOM mineralization, introduced as part of the BNI trait package. SOM mineralization rates were nearly doubled (*P* < 0.05) in root-zone soils of BNI-MUNAL compared to MUNAL control within low to medium *N* treatments but not under high *N* treatment (250 kg ⋅ N ⋅ ha^−1^) ( *SI Appendix*, Fig. S9*C* ), further supporting such a hypothesis. Nevertheless, the potential impact of BNI function on SOM mineralization beyond root-zone soils (i.e., bulk soils) remains unknown at this stage.

Enhanced BNI production from BNI-MUNAL root systems decreased soil NO_3_^−^ levels and improved soil NH_4_^+^ availability in field plots. Also, the enhanced GSA in leaves and lower natural abundance (δ^15^N in ‰) values (*P* < 0.002) in wheat grain ( *SI Appendix*, Fig. S9*D* ) indicated the likelihood of higher ammonium assimilation. Lower δ^15^N values indicate reduced soil microbial nitrification, a process known for strong isotopic N fractionation, which results in ^15^N enrichment of source N (soil NH_4_^+^) (39, 40). *Brachiaria* grass genotypes that have a high BNI capacity in root systems show lower δ^15^N in leaf tissues compared to low BNI capacity genotypes (41). Lower grain δ^15^N observed in BNI-MUNAL, 16% lower (*P* < 0.002) than in MUNAL control ( *SI Appendix*, Fig. S9*D* ), suggested reduced nitrification and consequently lower NO_3_^−^ availability and improved NH_4_^+^ assimilation during the growth period. In addition to yield, the introduction of T3BL.3Ns^b^S into MUNAL and ROELFS did not affect the grain protein profile, protein levels, or breadmaking attributes ( *SI Appendix*, Table S8 *A* and *B* and Fig. S10) and therefore should facilitate a broader deployment of BNI traits in wheat production.

### BNI Trait Introduction Did Not Negatively Impact Wheat Yield Potential.

GYs of BNI-MUNAL were either akin to MUNAL control (field trials of Obregon-1 and Obregon-2 in 2020 and in Obregon-1 in 2019 in which soils are of coarse sandy clay type, with a soil pH of 7.7) or significantly (*P* < 0.05) higher (in Obregon-2 in 2019) ( *SI Appendix*, Study 6c and Table S9) under high N input systems (≥250 kg ⋅ N ⋅ ha^−1^). However, for BNI-ROELFS, GYs were significantly (*P* < 0.05) lower than ROELFS control in three of the Obregon trials and were not significantly different in one trial ( *SI Appendix*, Table S9). It should be noted that BNI-ROELFS is at the BC_5_ stage, whereas BNI-MUNAL is at the BC_6_ stage. These results suggest that introduction of a BNI trait into these elite wheats did not negatively impact yield potential under high N inputs, even though soil conditions (i.e., because of alkaline soil pH) were not best suited for optimum BNI trait expression (5, 6, 23–26).

## Discussion

The last five decades of wheat breeding did not result in improvements in the BNI capacity of root systems, thus necessitating the use of wild grasses to source this trait. Two perennial wild grasses, *L. racemosus* and *L. mollis*, were identified as potential donors for a BNI trait. During this study, we showed the feasibility for transferring a chromosome arm controlling BNI traits (T3BL.3Ns^b^S) into modern wheats without disrupting their agronomic performance, grain protein content, or breadmaking quality ( *SI Appendix*, Tables S7 and S8 *A* and *B* and Fig. S10 *)*. Introducing a BNI trait into MUNAL resulted in substantial improvements in *N* uptake and significant yield gains across *N* inputs, particularly under low nitrogen inputs. The potential for further genetic gains in enhancing BNI capacity is evident from reducing the size of T3BL.3Ns^b^S (e.g., T3BL.3Ns^b^S-Tr-3 and T3BL.3Ns^b^S-Tr-7). Also, *L. mollis* can provide an additional genetic platform for introducing BNI traits into cultivated wheat. These field studies are part of establishing a proof of concept of trait expression in the field and potential beneficial impacts. Further studies are needed in agricultural settings, using multilocations with varying *N* inputs, to assess elite BNI wheats’ productivity gains. Similarly, future research should investigate how the BNI trait interacts with diverse environmental conditions (e.g., soil moisture, soil texture, SOM levels) as part of identifying farmlands suitable for deploying BNI wheats.

A major shift to “all nitrate” nutrition is the hallmark of modern production systems (6, 7) and is largely responsible for *N* pollution reaching the present crisis (42, 43). By inhibiting nitrifier activity, BNI wheats can facilitate dual *N* form availability in the soil root zone, which is more conducive for plant growth and can possibly enhance yield potential. Dual *N* forms provide better optimization of internal *N* assimilation pathways compared to all NO_3_^−^ nutrition (15–17, 44). In addition, BNIs may promote mycorrhizal associations (45, 46), which can further improve N and P uptake. The enhanced NH_4_^+^ assimilation and the resulting rhizosphere acidification can bring additional benefits, such as improving BNI release further through positive autofeedback regulation and enhanced micronutrient availability (23, 26, 47, 48). Interplay between these cascading sequences of interconnected rhizosphere processes and their accrued synergistic interactions can result in improved plant growth and productivity (as observed in BNI-MUNAL across *N* treatments of the field study in Japan 2019).

In principle, BNI traits can be introduced into elite wheat varieties that fit into various production systems globally, although it is likely that light-textured soils with a pH < 6.0 are more suited than alkaline heavy clay soils for BNI trait expression (23–26, 47, 48). Both spring and winter wheat types can benefit from BNI trait introduction. Assuming that the expression of a BNI trait is similar in winter wheats, the longer growing season may generate a higher inhibitory impact and be more effective. Large-scale yield trials with BNI wheats suggest no yield penalty from BNI trait introduction in high *N* input systems. Thus, T3BL.3Ns^b^S can be transferred into wheat varieties adapted to diverse geographical regions to improve the BNI capacity of cultivated wheats. In addition, the increasing CO_2_ levels in the atmosphere favors assimilation of NH_4_^+^ over NO_3_^−^ in wheat (49), underlining the importance of shifting *N* nutrition in wheat systems away from NO_3_^−^ and toward more NH_4_^+^.

## Conclusions and Perspectives

We have demonstrated the feasibility of enhancing BNI capacity in elite wheats by transferring a chromosome arm 3Ns^b^S controlling BNI traits from wild grass as a wheat *L. racemosus* translocation chromosome (T3BL.3Ns^b^S). The enhanced BNI release from root systems of T3BL.3Ns^b^S (about two- to fivefold increase) resulted in the suppression of soil nitrifier activity, reduced levels of soil nitrates, enhanced soil NH_4_^+^ availability, and accelerated SOM mineralization, which led to substantial gains in *N* uptake and GY. Also, the introduction of BNI traits led to major changes to *N* metabolism, indicating a shift toward more NH_4_^+^ assimilation. The newly developed BNI elite wheats retained their yield potential with an enhanced ability to uptake N from SOM mineralization (as evident from 58% higher N uptake in BNI-MUNAL under no N treatment), which has implications for the adaptation to low N input systems.

Nearly 60% of wheat area is in the Global South (50), mostly rainfed and highly variable in soil N. With an anticipated shortage of irrigation water, the likelihood of wheat being grown in more marginally productive areas in the future is high. Considering current and future food security hotspots, strong BNI performance in low N growing environments would become even more valuable. It remains to be seen how the newly developed BNI wheats perform in real production environments, particularly under low to moderate N inputs but also under intensive farming with high N inputs because also here, BNI traits may facilitate productivity gains (51). Most developed countries agreed to implement the Paris Climate Accord, committing to reduce agricultural greenhouse gas emissions by 30% by 2050. They could be early adopters of BNI wheats in intensive production systems (52). From an agronomic perspective, the aim is to minimize nitrogen leakage, allowing for lower *N* fertilization without losing productivity. The success of BNI wheats will be judged on their ability to maintain or improve productivity with reduced *N* inputs. This will drive their adoption.

The positive impact from BNI wheats on soil *N* cycling processes (e.g., reduced NO_3_^−^ leaching, lower N_2_O emissions, and improved soil N retention) needs to be validated and quantified in field settings closer to farmer conditions. A comparative advantage of BNI wheats needs to be assessed based on operational-scale field plots in multilocations (representing wheat production environments) to understand the potential value (i.e., productivity gains while reducing *N* losses) of BNI trait introduction. Like most production technologies or plant traits, it is likely that BNI traits will function only in certain agro-ecosystems and soil types. Based on current knowledge, BNI function is best expressed under mild acidic soil conditions, which potentially limits areas where BNI wheats can be deployed with current technology. If BNI traits could, in the future, be sourced from other wild grasses that can express well under alkaline soils, BNI wheat deployment could be expanded.

The genetic exploitation of BNI capacity in root systems of staple crops has the potential to facilitate the development of low-nitrifying and low N_2_O–emitting production systems. Being a seed-based technology and representing a nature-based solution, the potential for BNI wheats’ greater scalability and wider adoption can be high (51). An integrated approach involving BNI-enabled crops with a strategic deployment of SNIs may be the most effective approach to make farming more *N* efficient and less *N* leaky. It is likely that SNIs will be more effective on BNI crops due to the complementary nature of these inhibiting compounds (i.e., BNIs and SNIs), which suppress different segments of nitrifier populations (e.g., BNIs are more effective on AOAs, while SNIs are more effective on AOBs). The results presented here may have the potential to usher in a new era of BNI-enabled elite wheats based on genetic resources of wild grass. Realizing the full potential of BNI wheats, however, depends on a successful transfer of BNI trait (T3BL.3Ns^b^S) into elite varieties adapted to diverse agro-climatic conditions representative of wheat growing areas. In our view, this requires a major initiative by the global wheat research community, private sector, policy makers, and research funders (51, 52).

## Materials and Methods

Several growth-chamber experiments ( *SI Appendix*, Study 1, Study 2a and b, Study 4, and Study 5) were conducted to characterize wheat genetic stocks for BNI capacity using hydroponically grown plants. Plants are grown for about 50 to 60 d, and root exudates are collected using aerated trap solutions (e.g., 0.5 mM NH_4_Cl + 200 mM CaCl_2_) for 24 h to collect BNI activity. Root exudates are evaporated to dryness, and BNI activity is extracted and determined using luciferous recombinant *Nitrosomonas* assay described earlier (22). The BNI activity of root exudates is expressed in ATU ⋅ g^−1^ root dwt. ⋅ d^−1^ (see *SI Appendix*, *Materials and Methods* for details of each experiment and genetic stocks involved). Detailed methodology involved in developing wheat alien addition/translocation lines are described in *SI Appendix*, Study 3 ( *SI Appendix*, *Materials and Methods* *)*. During 2019, two BNI isogenic MUNAL genetic stocks (MUNAL control and BNI-MUNAL) were evaluated at JIRCAS experimental station in Tsukuba, Japan, to validate the effectiveness of BNI traits in suppressing soil nitrification, nitrogen uptake, and productivity gains in a range of nitrogen inputs ( *SI Appendix*, Study 6a and *Materials and Methods* for detailed description of this field study). A soil microcosm study was undertaken with BNI isogenic MUNAL genetic stocks at the University of the Basque Country, Spain ( *SI Appendix*, Study 6b) to validate BNI impact on soil nitrifier populations using plants grown in pots in a greenhouse ( *SI Appendix*, *Materials and Methods* for details). The yield potential of BNI isogenic lines of MUNAL control, BNI-MUNAL, ROELFS control, and BNI-ROELFS were measured in the CIMMYT field station in Obregon, Mexico, during 2018 and 2019 ( *SI Appendix*, Study 6c and *Materials and Methods* for details).

## Supplementary Material

Supplementary File

## Data Availability

The genetic stocks developed and presented in this study are deposited with CIMMYT and available upon request subjected to consent from JIRCAS.
