# Supplementary material for: Enlisting wild grass genes to combat nitrification in wheat farming: A nature-based solution
Source: Proc Natl Acad Sci U S A. 2021 Aug 23;118(35):e2106595118. doi: 10.1073/pnas.2106595118 (PMC8536370; doi:10.1073/pnas.2106595118)
Supplement: Supplementary File [file pnas.2106595118.sapp.pdf]

Supplementary Information

Title:

Enlisting wild-grass-genes to combat nitrification in wheat farming: a nature-based solution

*Guntur V. Subbarao[a,1], *Masahiro Kishii[b], Adrian Bozal-Leorri[c], Ivan Ortiz-Monasterio[b], Xiang Gao[a], Maria Itria Ibba[b], Hannes Karwat[b], M. B. Gonzalez-Moro[c], Carmen Gonzalez-Murua[c], Tadashi Yoshihashi[a], Satoshi Tobita[d], Victor Kommerell[b], Hans-Joachim Braun[b] and Masa Iwanaga[a]

Email:subbarao@jircas.affrc.go.jp

This PDF file includes:

Materials and Methods
References Cited in the supplementary 'Materials and Methods'
Figures S1 to S10
Tables S1 to S9

Materials and Methods

Study-1. BNI-capacity characterization of wheat varieties released from 1950-2010

Growing plants hydroponically in growth chamber

Twenty wheat varieties (Table S1) are grown hydroponically (using 70 L tanks) for 46 days in a walk-in growth chamber with day/night temperature regime of 24/20°C, photoperiod 13/11 h (light/dark) with a light intensity averaging of about 300 mmol m^{-2} s^{-1}; relative humidity is maintained at 70%. Each tank has a styrofoam top with eight holes in two rows; each genetic stock is planted four plants (14 days old) per hole and in four holes; in total 16 plants for each genetic stock and four plants are used for collecting root exudates and considered as one replication. There were four replications for each genotype. Plants are grown in full-strength nutrient solutions with 2 mM N as NH_4NO_3 for 37 days from planting (DAP) as described earlier (1); pH of nutrient solutions are adjusted to 6.0 – 6.5 once in a day. Nutrient solutions in tanks are replaced with fresh nutrient solutions once in every week. From 37 DAP, the nitrogen source is changed to 2 mM N as $(NH_4)_2SO_4$ and plants are grown until 42 DAP before using them for collecting root exudates.

Root exudates collection and BNI activity determination

Intact wheat plants of 42 days old are removed from solution tanks, rinsed with deionized water for a few minutes and transferred to 2 L bottles containing 1.8L of aerated solutions of 0.5 mM NH_4Cl + 200 μM $CaCl_2$ for 24 h, followed by another 24 h root exudate collection using 1.0 mM NH_4Cl + 200 μM $CaCl_2$. Four plants are used for each genetic stock for collecting root exudates and is considered as one replication. Four replications are used for each genotype for collecting root exudates. Root exudates from two collections for each replication are pooled, evaporated to dryness using a rotary-evaporator at 45°C. The residue is extracted with 20 mL methanol. The methanol extract is further evaporated to dryness and extracted with dimethyl-sulfoxide (DMSO), which is used for determining BNI activity using luciferous recombinant *Nitrosomonas* as described earlier (1). The BNI activity of root exudate is expressed in ATU g^{-1} root dwt. d^{-1} (*SI Appendix, Table S1*)

Study-2a. Characterization of *L. racemosus* for BNI-capacity

L. racemosus (*Lam*) Tzvelev (2n=4x=28; genome Ns NsXmXm) were grown from vegetative cuttings in pots filled with 13 kg of Andosol (Typic Hapludands, soil pH 5.5) for about one year before using them for collecting root exudates. Intact plants with roots were removed from pots and the soil attached to roots was removed and considered rhizosphere soil, which was used for soil incubation studies for determining nitrification rates. After removing the attached soil from roots, the roots were washed with running tap water initially and finally with deionized water before using them for collecting root exudates using the following root exudate collection solutions (trap solutions) as follows.

1. 1st day of root exudate collection – aerated solutions of 0.5 mM NH_4Cl + 200 μM $CaCl_2$ for 24h
2. 2nd day of root exudate collection – aerated solutions of 1.0 mM NH_4Cl + 200 μM $CaCl_2$ for 24h
3. 3rd day of root exudate collection – aerated solutions of 200 μM $CaCl_2$ for 24h

Root exudates of three collections are pooled, and evaporated to dryness, extracted with methanol, and further processed as described earlier for other experiments. The root exudate residue is extracted with DMSO (dimethylsulfoxide) and BNI activity was determined using a recombinant luminescent *Nitrosomonas* as described earlier (1). The BNI activity of root exudate is expressed in ATU d^{-1} $plant^{-1}$ (Fig. 1b)

Study-2b. Characterization of amphiploids derived from wild species for BNI-capacity

Growing plants hydroponically in growth chamber

The amphiploid genetic stocks (*SI Appendix, Table S2a,b*) were grown hydroponically in a walk-in growth chamber and the experiment was replicated four times. Plant-growing conditions and the collection of root exudates, the processing of root exudates for BNI-activity extraction and determination are the same as the above experiments and results are presented in (*SI Appendix, Table S2c*).

Study-3. Bread Wheat and alien addition translocation lines - Plant Materials

Bread wheat and alien chromosome addition/translocation lines used in this study are summarized (*SI Appendix, Table S2d*). Lr#n-SA was transferred into five different CIMMYT wheat lines by sequential backcrossing five to seven times. Wild species and wheat-wild species complete or partial amphiploid lines were summarized in (*SI Appendix, Table S2a*). The first nine amphiploids (used in this study) were developed at CIMMYT or with collaboration of Utah in 1980-2000's except *T.Tur/E.Mollis* which was donated from Dr. Merker Arnulf (Swedish University of Agricultural Sciences) in 1980. The remaining 11 amphiploids evaluated during this study were obtained from Prof. Bikram Singh Gill, KSU, USA (*SI Appendix, Table S2b*).

3a. The production of Robertosonian/centromeric and recombinant translocation lines of Lr#n

To produce centromeric translocation lines, the disomic addition line of Lr#n was firstly crossed with the 3B or 7B chromosome monosomic lines (cv. Chinese Spring background; Mono 3B CS; 2n=41=AABBDD) (*SI*

Appendix, Fig. S4a). The development of the addition lines and homoeology group of Lr#n *L. racemosus* chromosomes were described in previous studies (2-3). The chromosome constitution of F1 plants were 2n=42 or 43. The plants of 2n=42 were selected using carmine-stained chromosome counting. The presence of Lr#N was further confirmed by GISH. During the meiosis, spindle fibers will attach to both sides of univalent chromosomes of 3B and Lr#N, which then causes chromosome breakages in the centromeric regions at some frequency. The broken chromosome arms are sticky without telomere protection and may fuse each other to become centromeric translocations (*SI Appendix, Fig. S4a)*. The presence of a half arm translocation in F2 progenies was identified by GISH and FISH.

Further reduction of Lr#n-SA was done using *ph1b* mutant which induced recombination between wheat and alien chromosomes and has been heavily used for this purpose in the last several decades (4). CS *ph1b* was crossed with Lr#n-SA and progeny plants with homologous ph1b and heterologous Lr#n-SA were selected by GISH and a PCR marker gwm213 (gwm213-F: TGCCTGGCTCGTTCTATCTC; gwm213-R: CTAGCTTAGCACTGTCGCCC) (*SI Appendix, Fig. S5)*. Tr#3 spontaneously developed without using *ph1* mutant.

***In situ* hybridization**

Chromosome preparation and FISH/GISH analysis were conducted by the procedure of Dou et al. (5) with minor modifications. Genomic DNA of *L. racemosus* was labelled with a fluorescence molecular, tetramethyl-rhodamine-5-dUTP (Roche) using random primer method. For FISH analysis, 5 end fluorescein-12-dUTP (FICT) labelled oligonucleotide probes of Oligo-AAG (FITC-$(AAG)_{10}$) and Oligo-pLrtaiI-1 (FITC-CTGATTATAGACATTAGTCCCCCTGGTAGGGACAAACACAGCTTTCTGCAGATGCTCCAT) were obtained from SIGMA. Oligo-AAG makes it possible to identify all wheat B genome chromosomes (6), and Oligo-pLrtaiI-1 can stain TaiI family sequences which is present in the short-arm end of Lr#n chromosome (7).

C-banding

C-banding to identify all 42 wheat chromosomes was conducted the procedure of William and Mujeeb-Kazi (8). Root tips were fixed in 0.1 aceto-carmine for 48 hours and squashed in 45% acetic acid on slides. The chromosomes on slides were stained by immersing the slides in barium hydroxide [$Ba(OH)_2$] solution.

Study-4. Characterization of *L. racemosus*-CS translocations for BNI-capacity

4a. 1st experiment

Five *L. racemosus*-CS translocations that include CS, Lr#n addition, Lr#n-SA translocation on 7B, Lr#n-SA translocation on 3B, and Lr#n-LA (long-arm translocation) (Table 1) are evaluated for BNI-capacity. Plants are grown hydroponically for 60 days as described earlier in a walk-in growth chamber as described above for experiments 1-3 and the experiment is replicated four times. Root exudates are collected and processed for BNI-activity as described earlier and results are presented in Table 1.

4b. 2nd experiment

Four genetic stocks that include two *L. racemosus*-CS shortened short-arm of Lr#n translocations along with CS and Lr#n-complete short-arm translocation (Table 2) were evaluated for BNI-capacity. Plants were grown hydroponically using a walk-in growth chamber and growing conditions are the same as described earlier. The only difference is that plants were grown for 49 d (instead of 42 DAP for earlier studies) in nutrient solutions before using them for root exudate collection, i.e. plants were seven days older than those in earlier experiments. Root exudates collection and processing for BNI-activity extraction and determination is same as described for earlier experiments and results are presented in Table 2.

Study-5. Characterization of Lr#n-SA translocations (T3BL.3Ns^{b}S) in elite-wheats for BNI-capacity

5a. 1st experiment

Eight genetic stocks representing elite-wheat translocations (Table 3) were evaluated for BNI-capacity in root systems. Plants were grown hydroponically in a walk-in growth chamber for 42 days in nutrient solutions as described earlier. Root exudates are collected using 42 d old intact wheat plants and four replications for each genetic stock. Root exudate collection protocol, BNI-activity extraction and BNI activity determinations were the same as before and results are presented in Table 3.

5b. 2nd experiment

Two genetic stocks (representing BNI-isogenic lines of MUNAL-control and BNI-MUNAL (Lr#n-SA) were evaluated extensively for BNI-capacity in root systems. Plants are grown hydroponically in a walk-in growth chamber for 30 days (30 DAP) before collecting root exudates. Growing conditions are the same as for the earlier experiments except that the pH of the nutrient solutions adjusted to 5.0 to 5.5 during growth period (unlike 6.0 to 6.5 pH in earlier experiments). Plants are grown in nutrient solutions as described earlier using NH_4NO_3 as N source for three weeks. From 3rd week onwards, the nitrogen source changed to 1 mM N as $(NH_4)_2SO_4$ for one

week (i.e. last week of plant growth) before using them for collecting root exudates. Also, plants are grown only for 30 DAP unlike 42 DAP for earlier studies.
Root exudates are collected using intact wheat plants using 1.8 L aerated solutions (in 2L black plastic bottle) of the following compositions as follows:

1. RE-NH_4-1 (1.8L of aerated solutions of 0.5 mM NH_4Cl + 200 mM $CaCl_2$ for 24h – 1st day)
2. RE-nutri-NH_4-1 (1.8L of aerated solutions of 1/4th strength nutrient solution with 0.5 mM NH_4Cl for 24h – 2nd day collection)
3. RE-water-1 (1.8L of aerated solution of 200 mM $CaCl_2$ for 24h – 3rd day collection)
4. RE-NH_4-2 (1.8L of aerated solutions of 1.0 mM NH_4Cl + 200 mM $CaCl_2$ for 24h – 4th day collection)
5. RE-nutri-NH_4-2 (1.8L of aerated solutions of 1/4th strength nutrient solution with 1.0 mM NH_4Cl for 24h – 5th day collection)
6. RE-water-2 (1.8L of aerated solution of 200 mM $CaCl_2$ for 24h – 6th day collection)

Root exudate samples are stored at 5 ◦ C before processing for extraction and determination of BNI activity as described earlier and the results are presented in Fig. 4.

Study-6a. Field validation of the effectiveness of BNI-trait (T3BL.3Ns^{b}S) in suppressing soil nitrification, nitrogen uptake and productivity gains in a range of nitrogen inputs using BNI-isogenic lines of MUNAL (MUNAL-control and BNI-MUNAL) – Field study in JIRCAS fields, Tsukuba, Japan.

A field experiment was established on the second week of November 2019 at JIRCAS experimental station in Tsukuba, Japan (36.05N 140.08E), on a volcanic ash soil (Typic Hapludands) [soil pH 5.5 (1:2.5 soil:water), clay 54.8%, silt 26.3%, sand 18.9%, total carbon = 29.2 mg C g^{-1} soil; total N = 2.5 mg N g^{-1} soil; and C/N ratio 11.7]. 100 kg P as SSP and K as K_2SO_4 as basal application at the time of field preparation. The entire field site was mapped for inorganic-N levels based on core soil samples taken at a depth of 0-20 cm. At least four soil samples were taken from each experimental plot. Inorganic-N levels were about 5 mg N kg^{-1} soil (NO_3^- + NH_4^+) in field plots. This field-site is under normal field management operations and continuously used for various experiments. These fields are not heavily fertilized but are well maintained and have good soil-fertility in general.

Two BNI-isogenic MUNAL-genetic stocks (MUNAL-control and BNI-MUNAL) with three N-treatments (0 kg N, 100 kg N ha^{-1}, 250 kg N ha^{-1}); N-fertilizer was applied in three equal splits (1st N application was on 11th December 2019; 2nd N application was on 24th March 2020; 3rd N-application was on 14th April). The experimental design is split-plot with N treatment as its main plot and genetic stocks as its subplot; the experiment was replicated four times. Four seedbeds with 1.5 m length (with 0.6 m space between two seedbeds) and 30 cm width formed one experimental unit which is 3 m wide and 1.5 m long in total. Two rows of wheat plants (with 10 cm spacing between plants) are planted in each seedbed. In total there were eight rows of wheat plants for each experimental unit. Four seeds were planted in each hole and two seedlings were grown up to maturity from each hole giving a plant density of 70 plants m^{-2}. Nitrogen was applied as ammonium sulphate solutions to the experimental plots to have uniformity in nitrogen distribution. The crop was harvested at maturity during July 2020. For the determination of total biomass production and grain yield, the middle two seedbeds of 1 m length (80 plants in total for each experimental plot) were harvested. After separating the grain, the remaining dry matter was oven dried at 70 ◦ C for 72 h and dry weights were recorded.

Soil core sampling
The second split of nitrogen fertilizer application was done on 24th March 2020. The soil core sampling was done on 31st March 2020 (i.e. seven days after the application of the second dose of N fertilizer). Four soil cores from each seedbed close to plant bases were collected at a depth of 0-20 cm. One seedbed was used for the first soil-core sampling. Soil samples from all four core samplings were pooled to make one composite sample for each replication and these results are presented in (*SI Appendix, Table S4*). The second core-soil sampling was done on 21st April (i.e. seven days after the application of the third split of N fertilizer on 14th April 2020) and the second seedbed was used for this sampling.

Leaf sampling for determination of NRA, GS and leaf nitrate levels
After the second split of N application (i.e. 24th march), leaf samples are collected on 3rd day, 7th day, and 9th day (1st sampling; data is presented only for 7th day). For collecting leaf samples, the most fully expanded leaf sample from 4 plants from each experimental plot and immediately stored in an icebox before analyzing them for NRA and GSA. Part of the leaf samples are stored at -20 ◦ C for determining leaf nitrate levels. Similarly, leaf samples are also collected after the 3rd split of N application (14th April) in a similar manner (i.e. 3rd day, 7th day and 9th day – 2nd sampling) for NRA, GSA and leaf nitrate level determinations. NRA and GSA are determined according

to Liu et al. (9); Karwat et al. (10). The data presented for NRA, GSA and leaf nitrate levels were all from leaf sampling done for 7th day from nitrogen fertilizer (ammonium sulphate solutions) application.

<u>*NRA determination in leaf tissue*</u>

For NRA determinations, 0.5 g of fresh tissue samples were ground in a pre-chilled mortar and pestle on ice in 4 mL of extraction buffer (pH 7.5) consisting of 25 mM K-phosphate, 10 mM cysteine, 1 mM EDTA. 1.0 mL of supernatant (after 10000 rpm for 10 min at 4 °C) was added to a reaction mixture containing 1.0 mL of 0.1 M K-phosphate buffer (pH 7.5) and 0.1 M KNO_3, and 0.5 mL 0.25 mM NADH. Controls were made by adding 0.5 mL of 0.1 M K-phosphate buffer (pH 7.5) instead of 0.5 mL 0.25 mM NHDH. The reaction took place at 25 °C for 60 min. The assay was terminated by the addition of 1% (w/v) sulphanilamide in 3 M HCl. Nitrite was determined with 1 mL 0.02% (w/v) *N*-naph-thyl-(1)-dihydrochloride. The color was then allowed to develop for 60 min at 25 °C before measuring the absorbance of supernatant (10000 rpm for 5 min) at 540 nm (Biotek Instruments, Vermont, USA). NR activity was expressed as mmol NO_2^- -N g^{-1} protein h^{-1}.

<u>*GSA determination in leaf tissue*</u>

GSA in leaves was determined based on Liu et al. (9); 0.5 g of leaf tissue was ground in a pre-chilled mortar and pestle on ice in 4 mL buffer solution (pH=8.0) containing 50 mM Tris-HCl, 2mM DTT, 2mM EDTA, 2 mM $MgSO_4$ $7H_2O$. 1.0 mL of supernatant (after 10000 rpm for 10 min at 4°C) was added to a reaction mixture (pH=7.8) containing 1.0 mL (containing 0.25 mM imidazole, 250 mM $MgSO_4$ $7H_2O$, 80 mM glutamate sodium, 20 mM L-cysteine, 2 mM EGTA), 0.5 mL 80 mM hydroxylamine hydrochloride, 0.7mL 30 mM ATP solution was added and incubated for 30 min at 37°C; a blank was prepared without hydroxylamine hydrochloride. The reaction was stopped by adding 1mL a reagent containing 0.2 mM TCA, 0.37 M $FeCl_3$ $6H_2O$, 0.6 M HCl. After 30 min, homogenate was centrifuged at 10000 g for 5 min, and the supernatant was using spectrophotometer at 540 nm (Biotek Instruments, Vermont, USA). The unit of GSA was the amount of enzyme catalyzing the formation of 1 mmol of glutamyl-monohydroxamate per hour. Only results from 1st sampling were presented for NR and GS activities in leaves (*SI Appendix, Table S6*).

<u>*Leaf nitrate determination*</u>

Leaf nitrate levels are quantified according to Karwat et al. (10). One g of (freeze-thawed) leaf tissue was ground with 50 mL of deionized water using a blender and the mixture was subjected to centrifuge, and the supernatant was used for nitrate analysis by auto analyzer (BL-TEC K.K. Tokyo, Japan). Leaf nitrate levels for both the 1st and 2nd samplings is presented in *SI Appendix, Table S5.*

<u>*Root-zone-soil sampling*</u>

Root-zone soil is defined as the soil that is in close proximity to roots (which includes soil that is attached to roots). On the 16th day after 2nd split of N application (i.e. 10th April), the first root-zone-soils collected from 4 plants. Intact plant roots were removed from the field. By gently shaking the roots, the loose soil that is in close proximity to roots that include soil that is attached to roots is removed and considered as root-zone-soil (*SI Appendix, Fig. S7a1*). The soil collected in this manner for four plants were pooled for each experimental plot (about 200 g in total) and treated as replication. Root-zone-soil was collected from all the experimental plots, i.e. four replications. A second root-zone-soil collection was made in a similar manner on 30th April (16 days after 3rd split of N application). The soil was air dried, sieved (2mm) and stored at 5°C until inorganic-N analysis, pH determinations, potential nitrification, and other laboratory soil incubation studies.

<u>*Determining potential soil nitrification*</u>

Potential soil nitrification was determined using a modified protocol of Hart et al. (11). The brief protocol is as follows: 10 g of root-zone-soil is taken into a 250 ml Erlenmeyer flask and 100 ml of N-potential solution was added; flask was covered with a breathable sponge-cap and loaded into a temperature-controlled roto-mixer at 250 rpm and incubated at 20°C; 10 mL of the soil slurry from flask was sampled at 24h intervals during a 10 d period. Soil slurry was subjected to centrifuge and the supernatant was analyzed for NO_2^- and NO_3^- using autoanalyzer (BL-TEC K.K. Tokyo, Japan). The results from potential soil nitrification in root-zone-soil is shown in *SI Appendix, Fig. S7a.*

<u>*Determining soil nitrification rates*</u>

The root-zone-soil was air dried and passed through 2-mm sieve and stored at 5°C in a cold room prior to using for nitrification studies as described earlier (1). The soil water status was maintained at 60% WFPS during the incubation period of 21days using a temperature and humidity-controlled incubator; incubation temperature was 20°C and RH was at 80%; two g of soil taken in a 10 ml glass bottle and 200 mg N kg^{-1} soil as $(NH_4)_2SO_4$ is added during the incubation period. The other details of the soil incubation study were described earlier (22). The results for potential-net-nitrification-rate and nitrate percentage of inorganic-N pool (%) in rhizosphere soil collected (from 1st sampling) is shown in Table 4.

<u>*Determination of nitrifier populations in root-zone-soils*</u>

Soil DNA was extracted from 0.4 g of root-zone-soil using Fast DNA Spin Kit for soil (Mo-Bio, Carlsbad, CA) according to manufacturer's instructions with slight modifications. The obtained pure DNA was quantified by the Qubit Quantification Platform dsDNA HS Assay kit (Invitrogen, Carlsbad, CA). Quantitative PCR (qPCR) was

performed to assess the abundance of the *amoA* genes of both ammonia-oxidizing bacteria (AOB) and ammonia oxidizing archaea (AOA). The detailed protocol was described in our earlier publication (12). The results for AOA and AOB populations in root-zone-soils (from 1st sampling) is shown in Fig. 5c; *SI Appendix, Fig. S7c.*

Determination of N_2O emissions in rhizosphere soils

Soil incubation studies are conducted using air-dried root-zone-soils from MUNAL-control and BNI-MUNAL following using Hink et al. (13). Root-zone-soils collected from 250 kg N ha^{-1} treatment plots were used for this study. Five g of air-dried soil was transferred to 100 mL glass vial (height 12.0 cm; diameter 3.5cm) and incubated with 250 mg N kg^{-1} soil N as $(NH_4)_2SO_4$ and soil moisture levels were maintained at 70% water holding capacity during a 21day incubation period. Glass vials with treatment soils were incubated in the dark at 20°C with 80% RH; glass vials were covered with parafilm during the incubation period. 24 h before the gas sampling, glass vials were covered with butyl rubber stopper and melamine white screw cap. After 24 h of closure, 30 mL of gas sample was taken from the headspace with a 50 ml syringe at different time intervals, 0-5, 7-11, 14, 17, 21 d incubation and were injected into 10 ml pre-evacuated vials. After gas sampling, all bottles were ventilated for 2 min, then sealed again with butyl rubber stopper or parafilm (depending on sampling schedule). Evaporated water was replenished with distilled water on weight basis. The N_2O concentration in the vials was measured with a gas chromatograph (GC-14B, Shimadzu, Tokyo, Japan) using an electron capture detector. The total net amount of N_2O fluxes over the sampling period was obtained by summing N_2O emission measure days (the N_2O concentration is about 0.378 ppm in the air). N_2O emission from root-zone-soils (1st sampling) is shown in Fig. 5b; *SI Appendix, Fig. S7b.*

Root-zone-soil pH and inorganic-N (NH_4^+ and NO_3^-) determinations

For root-zone-soil inorganic-N determinations, 3 g of soil was extracted with 30 mL of 2N KCl for 30 min using a shaker and the mixture is filtered. The filtrate is analyzed for NH_4^+ and NO_3^- using autoanalyzer (BL-TEC K.K. Tokyo, Japan). Soil pH was determined by a water soil ratio of 2.5:1. Results from root-zone-soil pH (for the 1st sampling) is presented in *SI Appendix, Fig. S7f.*

SPAD measurements for chrolorophyll concentrations

About 10 plants per experimental plot were selected for SPAD measurements using SPAD 502 plus (Konica Minolta, Japan); at least two readings from each plant were taken and the average of 20 measurements for each experimental plot was made and considered as representative value for the experimental plot. SPAD data presented in Fig. S8c comes from measurements taken on 7th day after 3rd split nitrogen fertilizer application. Wheat plants were heading during this sampling time.

Potentially mineralizable nitrogen content (PMN) analysis

PMN in root-zone-soil (1st sampling) is determined following Waring and Bremner (14) and Vazquez et al. (15) by anaerobic soil incubation during seven days at 37 ° C. The results for PMN in root-zone-soils is presented in *SI Appendix, Fig. S9c.*

$d^{15}N$ analysis of grain samples

$d^{15}N$ and N concentration in grain was determined using a Euro elemental isotope ratio mass spectrometer IRMS (Thermo Scientific, Bremen, Germany). The ^{15}N natural abundance of the sample relative to the standard (atmospheric N_2) was expressed as: $d^{15}N = [(R_{sample}/R_{standard})-1]x1000$ (‰), where R represents the isotope ratio ($^{15}N/^{14}N$) and $R_{standard}$ is $^{15}N/^{14}N$ for atmospheric N_2 that is 0.00368 ($d\ ^{15}N = 0$) (16). These results are presented in *SI Appendix, Fig. S9d.*

Study-6b. Soil-microcosm study at UPV-EHU, Spain

A greenhouse experiment was established on first week of September 2019 at the University of the Basque Country (UPV-EHU) in Leioa, Spain (43.32 N 2.96W) with a clay loam soil (Hypercalcic Kastanozem) [soil pH 8.0 in H_2O, clay 31.9%, silt 24.7%, sand 43.4%, organic matter 21.2 mg g^{-1} soil; 1.6 mg total N g^{-1} soil; and C:N ratio 8.15]. BNI-Isogenic lines of MUNAL-control and BNI-MUNAL were sown in 1.5L pots and grown in a greenhouse under a daily regimen of 14/10 h of light/darkness respectively, with an average day/night temperature of 25/18 ° C and relative humidity of 50/60%. Nitrogen was applied as ammonium sulphate in a single application at an equivalent dose of 200 kg N ha^{-1}. Soil moisture was adjusted at 60% water-filled pore space (WFPS) during the 30 d growth period. Soil samples were taken 30 days after N fertilization; soil mineral N measurements, DNA extraction and qPCR analysis were done according to Torralbo et al. (17). These results are presented in SI Appendix, Table S4b.

Study-6c. Field experiments in Mexico for yield potential evaluations on BNI-wheats

Yield potential of BNI-isogenic lines of MUNAL-control, BNI-MUNAL, ROELFS-control, and BNI-ROELFS were measured in Obregon, Mexico (27°24'N,109°5'W, 38m) in two growing seasons 2018/2019 and 2019/2020. The crop was sown in the end of November and harvested approximately five months later in the end of the following April. The soil type of the field station is a Coarse Sandy-Clay and the soil pH is 7.7 (10). The plot size

was 4 m × 1.6 m, with two replications. All nitrogen was applied at the time of sowing with the rate of 250 kg N ha^{-1}. These results are presented in *SI Appendix, Table S9.* These fields are part of CIMMYT experimental station, managed by the wheat breeding programs and are well fertilized. The grain yields reported here are within the range expected for 'MUNAL' in this region.

Study-6d. Analysis of grain quality parameters

Grain protein (GPRO, 12.5% moisture basis) content was measured using near-infrared spectroscopy (DA 7200 NIR, Perten Instruments, Sweden), with a calibration validated using Leco®/Dumas method (correction factor: 5.83. Equipment FP828 Leco Instruments, St Joseph, Michigan, USA). The grain samples were tempered with water according to the official AACC method 26-95 (18) and milled using a Brabender Senior mill (C.W. Brabender OHG, Germany). The obtained flour was used for the sodium dodecyl sulfate (SDS) sedimentation test (SDSS), according to the modified protocol described in Peña et al. (19). Flour protein (FPRO) (14% moisture basis) was determined using FT-near infrared spectroscopy (Antaris II, Thermo Scientific, Massachusetts, USA) calibrated based on Leco®/Dumas method (correction factor: 5.70. Equipment FP828 Leco Instruments. St. Joseph, Michigan, USA). The bread baking test was performed according to the AACC method 10-09 (13) using 100 g of refined flour. Bread loaf volume (LV) was determined by rapeseed displacement (AACC 10-05.01). Bread crumb structure was rated by an expert baker using values ranging from very good to poor.

HMW-GS and LMW-GS extraction and separation

The glutenin fraction was obtained according to Singh et al. (20) with some modifications. Specifically, 20 mg of whole-meal flour was mixed (1400 rpm) with 0.75 ml of 50% propanol (v/v) for 30 min at 65°C in a Thermomixer Comfort (Eppendorf). The tubes were centrifuged for 2 min at 10000 rpm, and the supernatant containing the gliadins was discarded. The same process was repeated twice to remove any remaining gliadins. The remaining pellet was then mixed with 0.1 ml of a 1.5% (w/v) DTT solution obtained with 50 µL of propanol at 50% and 50 µL of Tris-HCl 0.08M pH 8.0. The mixture was then mixed in a Thermomixer for 30 min at 65°C and 1400 rpm and centrifuged for 2 min at 10000 rpm. A volume of 0.1 ml of a 1.4% (v/v) vinylpyridine solution obtained with 50 µL of propanol at 50% and 50 µL Tris-HCl 0.08M pH 8.0, was added to the tube, mixed in a Thermomixer for 15 min at 65°C and at 1400 rpm, and finally centrifuged for 5 min at 13000 rpm. The supernatant containing the glutenins was then transferred to a new tube and mixed with the same volume of extraction buffer (Tris-HCl 0.1M, 2% SDS (w/v), 40% glycerol (w/v), and 0.02% (w/v) bromophenol blue, pH 6.8) and incubated in the Thermomixer for 5 min at 90°C and 1400 rpm. The tubes were centrifuged for 5 min at 10000 rpm, and 6 µl of the supernatant were used to load the gels. Glutenins were separated in polyacrylamide gels (15% T) prepared using 1M Tris buffer, pH of 8.5. Gels were run at 12.5 mA per gel for about 20 h. The Glu-1 and Glu-3 alleles were classified using the systems developed by Jackson et al. (21) and Branlard et al. (22). Seed protein quality and breadmaking quality results are presented in *SI Appendix, Table S8.*

References Cited in the supplementary 'Materials and Methods'

1. G. V. Subbarao, T. Ishikawa, O. Ito, K. Nakahara, H. Y. Wang, W. L. Berry, A bioluminescence assay to detect nitrification inhibitors released from plant roots: a case study with *Brachiaria humidicola*. *Plant Soil* **288**, 101-112 (2006).
2. O. U. Edet, J. S. Kim, M. Okamoto, T. Takeda, M. Kishii, Y. S. A. Gorafi, H. Tsujimoto, Efficient anchoring of alien chromosome segments introgressed into bread wheat by new *Leymus racemosus* genome-based markers. *BMC Genet* **19**, 18 (2018), doi: 10.1186/s12863-018-0606-1.
3. M. Kishii, T. Yamada, T. Sasakuma, H. Tsujimoto, Production of wheat-*Leymus racemosus* chromosome addition lines. *Theor Appl Genet* **109**, 255-260 (2004).
4. E. R. Sears, An induced mutant with homoeologous pairing in common wheat. *Can J Genet Cytol* **19**, 585-593 (1977).
5. Q. W. Dou, H. Tanaka, N. Nakata, H. Tsujimoto, Molecular cytogenetic analysis of hexaploidy lines spontaneously appearing in octoploid Triticale. *Theor Appl Genet* **114**, 41-47 (2006).
6. A. Cuadrado, T. Schwarzacher, N. Jouve, Identification of different chromatin classes in wheat using *in situ* hybridization with simple sequence repeat oligonucleotides. *Theor Appl Genet* **101**, 711-717 (2000).
7. M. Kishii, K. Nagaki, H. Tsujimoto, T. Sasakuma, Exclusive localization of tandem repetitive sequences in subtelomeric heterochromatin regions of *Leymus racemosus* (Poaceae, Triticeae). *Chromosome Res* **7**, 519-529 (1999).

8. M. D. H. M. William, A. Mujeeb-Kazi, Biochemical and molecular diagnostics of *Thinopyrum bessarabicum* chromosomes in *Triticum aestivum* germplasm. *Theor Appl Genet* **90**, 952-956 (1995).
9. M. Liu, D. Tang, Y. Shi, Y. Li, Q. Zhang, J. Ruan, Short-term inhibition of glutamine synthetase leads to reprogramming of amino acid and lipid metabolism in roots of leaves of tea plant (*Camellia sinensis* L.). *BMC Plant Biol* **19**, 1-17 (2019).
10. H. Karwat, M. A. Sparke, F. Rasche, M. J. Arango *et al.*, Nitrate reductase activity in leaves as a plant physiological indicator of *in vivo* biological nitrification inhibition by *Brachiaria humidicola*. *Plant Physiol Biochem* **137**, 113-120 (2019).
11. S. C. Hart, J. M. Stark, E. A. Davidson, M. K. Fireston, Nitrogen mineralization, immobilization and nitrification. *In: Methods of Soil analysis, Part 2, Microbiological and Biochemical Properties* (pp. 985-1018). Madison, WI: Soil Science Society of America (1994).
12. P. S. Sarr, Y. Ando, S. Nakamura, S. Deshmpande, G. V. Subbarao, Sorgoleone release from sorghum roots shapes the composition of nitrifying populations, total bacteria, and archaea and determines the level of nitrification. *Biol Fertil Soils* **56**, 145-166 (2020).
13. L. Hink, C. Gubry-Rangin, G.W. Nicol, J. I. Prosser, The consequences of niche and physiological differentiation of archaeal and bacterial ammonia oxidisers for nitrous oxide emissions. *ISME J* **12**, 1084-1093 (2018).
14. S.A. Waring, J. M. Bremner, Ammonium production in soil under waterlogged conditions as an index of nitrogen availability. *Nature* **201**, 951-952 (1964).
15. E. Vazquez, N. Teutscherova, M. Dannenmann, P. Tochterle, K. Butterbach-Bahl, M. Pulleman, J. Arango, Gross nitrogen transformations in tropical pasture soils as affected by *Urochloa* genotypes differing in biological nitrification inhibition (BNI) capacity. *Soil Biol Biochem* **151**, 1-10 (2020).
16. A. R. Jones, R. C. Dalal, Enrichment of natural ^{15}N abundance during soil N losses under 20 years of continuous cropping. *Sci Total Environ* **574**, 282-287 (2017).
17. F. Torralbo, S. Menendez, I. Barrena, J.M. Estavillo, D. Marino, C. Gonzalez-Murua, Dimethyl pyrazol-based nitrification inhibitors effect on nitrifying and denitrifying bacteria to mitigate N_2O emission. *Scientific Reports* **7**, 1-11 (2017).
18. American Association of Cereal Chemists, *Approved Methods of the AACC*, St. Paul, MN, USA (2010).
19. R. J. Pena, S. Amaya, A. Rajaram, A. Mujeeb-Kazi, Variation in quality characteristics associated with some spring 1B/1R translocation wheats. *J Cereal Sci* **12**, 105-112 (1990).
20. N. K. Singh, K.W. Shepard, G.B. Cornish, A simplified SDS-PAGE procedure for separating LMW subunits of glutenins. *J Cereal Sci* **14**, 203-208 (1991).
21. E. A. Jackson, M. H. Morel, T. Sontag-Strohm, G. Branlard, E.V. Metakovsky, R. Redaelli, Proposal for combining the classification systems of alleles of *Gli-1* and *Glu-3* loci in bread wheat (*Triticum aestivum L.*). *J Genet Breed* **50**, 321-336 (1996).
22. G. Branlard, M. Dardevet, N. Amiour, G. Igrejas, Allelic diversity of HMW and LMW glutenin subunits and omega-gliadins in French bread wheat (*Triticum aestivum L.*). *Genet Resour Crop Evol* **50**, 669-679 (2003).

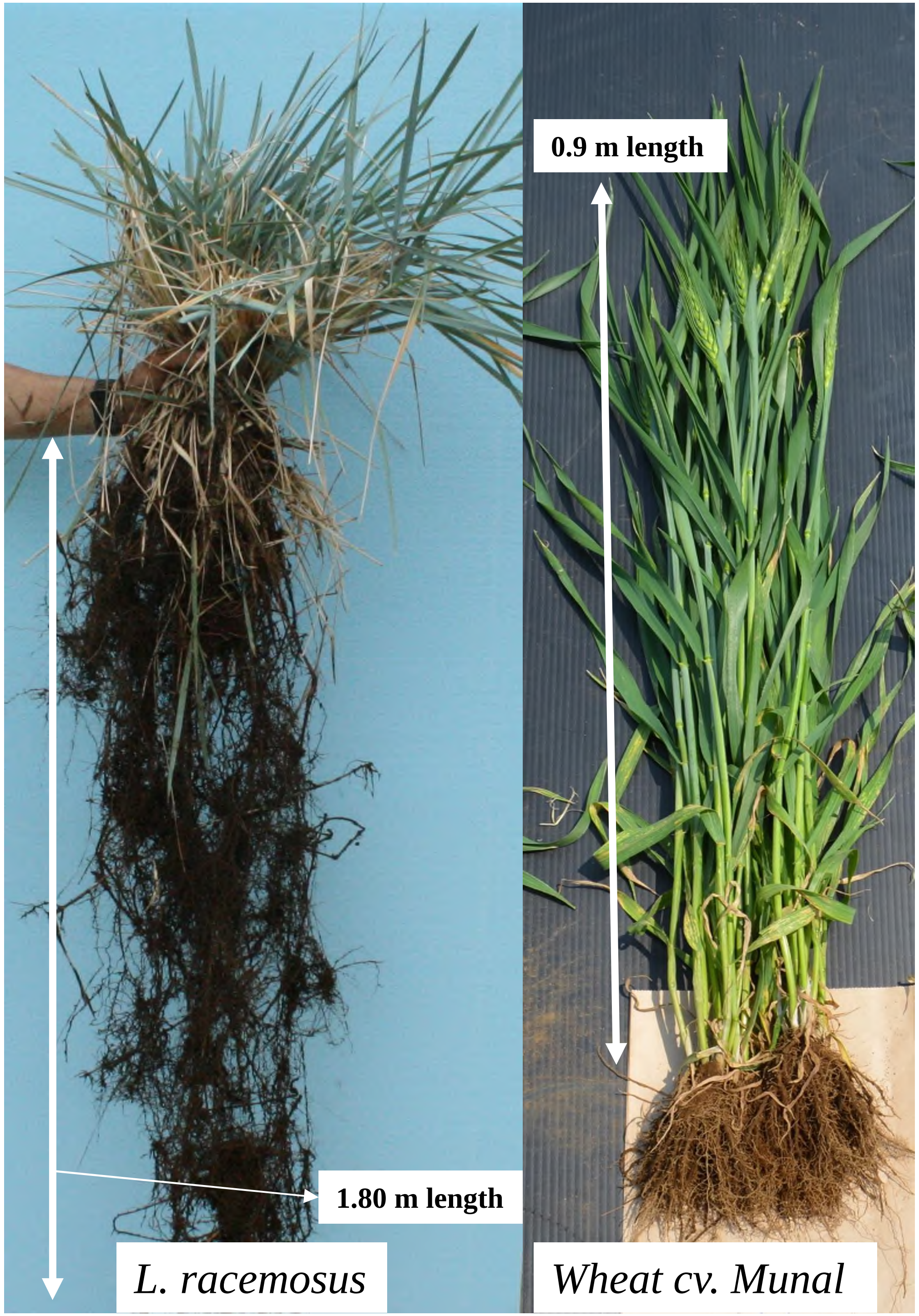

Fig. S1. Wild-grass (*L. racemosus*) forms extensive root system. plants in this picture are grown in 13 kg soil pots for about one year. Root systems in this picture are massive fibrous roots which are close to 2 m long (study-2a).

Cultivated wheat cv. MUNAL grown in the field with the same soil-type for 120 d (250 kg N ha^{-1} treatment field plots) (JIRCAS field study-6a; 2019-20)

**Serial No.**	**Treatment**	**Total inorganic-N (NH_4^++NO_3^-) in soil (ppm) at the end of incubation period**	**Nitrate percentage of inorganic-N pool (%)**	
		Mean	Mean	*SE*
**1**	**Control**	**583.5**	**97.1**	*0.4*
**2**	**Leymus-bulk soil**	**611.3**	**67.9**	*1.33*
**3**	**Leymus-rhizosphere soil**	**617.5**	**38.5**	*0.93*

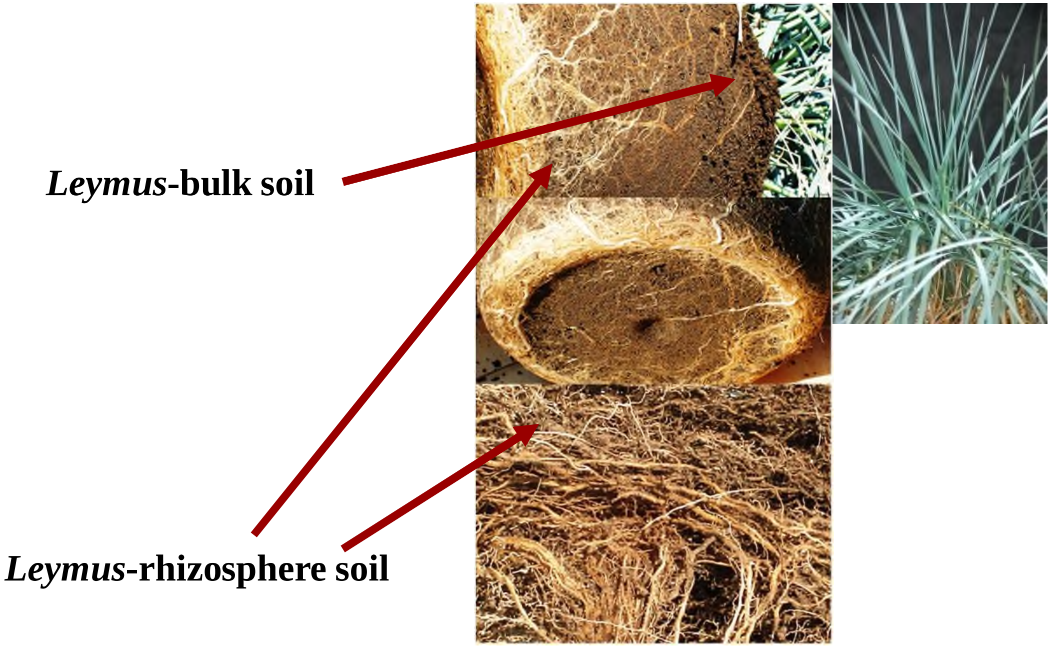

Fig. S2. Nitrate percentage of inorganic-N pool in *Leymus*-rhizosphere soil and *Leymus*-bulk soil and in control soil after 60 d incubation period using soil-microcosm study (Study-2a); Two g of air-dried soil samples were incubated with 400 ㎍ of NH_4^+-N [as $(NH_4)_2SO_4$] at 20 ° C with RH of 80% in a temperature-humidity controlled incubator for 60 d period before determining nitrification rate. Soil incubation protocol as described earlier (22). *Leymus* plants are grown in 13 kg soil pot^{-1} for about one year before using rhizosphere soils and bulk soil from the potted plants for this soil-microcosm study; control soil is the same soil maintained in a separate pot without plants but watered and fertilized in the same way as the *Leymus* planted soil in the pot. Soil nitrification (%) = [soil-NO_3^- /(soil-NO_3^- + soil-NH_4^+) X 100]. (*Values are means* $\pm$ *SE of four replications).*

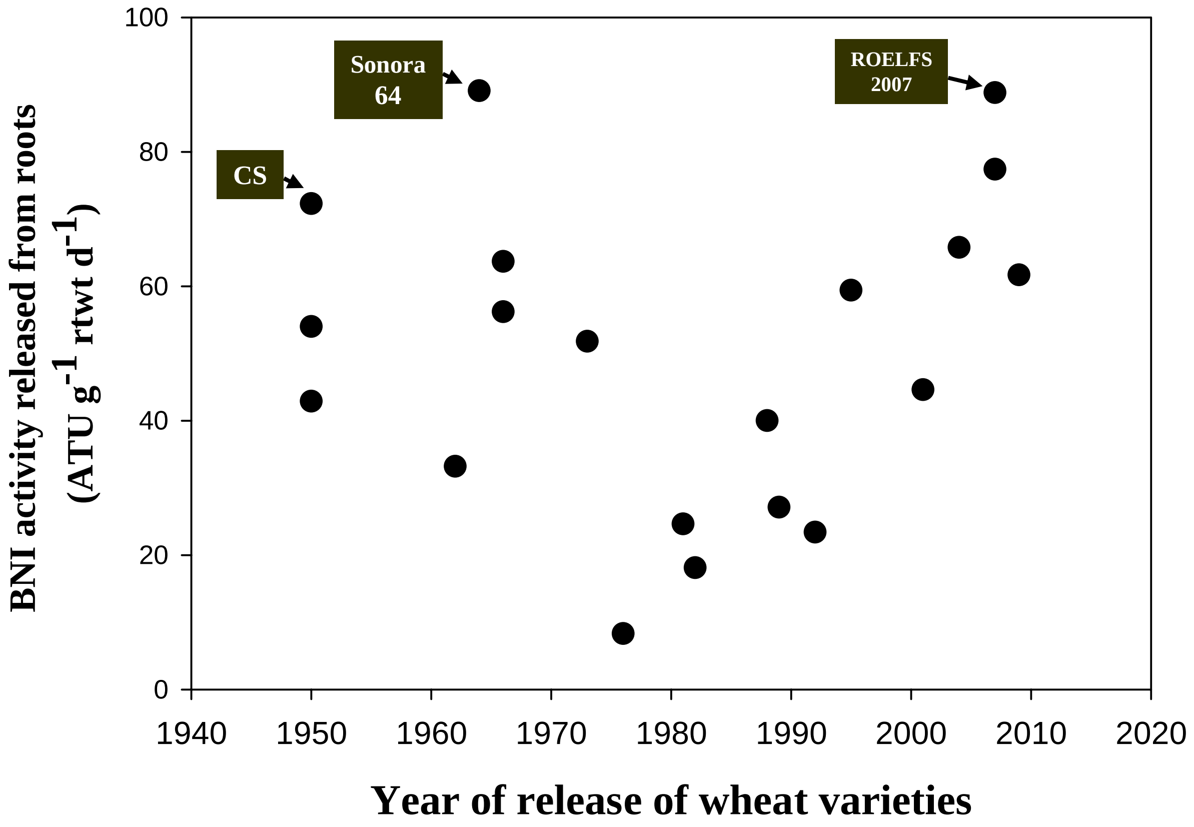

Fig. S3. Year of wheat varieties released and their BNI-capacity in root systems (based on Study-1). These results suggest no relationship between wheat breeding under high-N input conditions to BNI-capacity in root systems (Study-1; Based on the data presented in Table S1 (*Each data point is mean of four replications*).

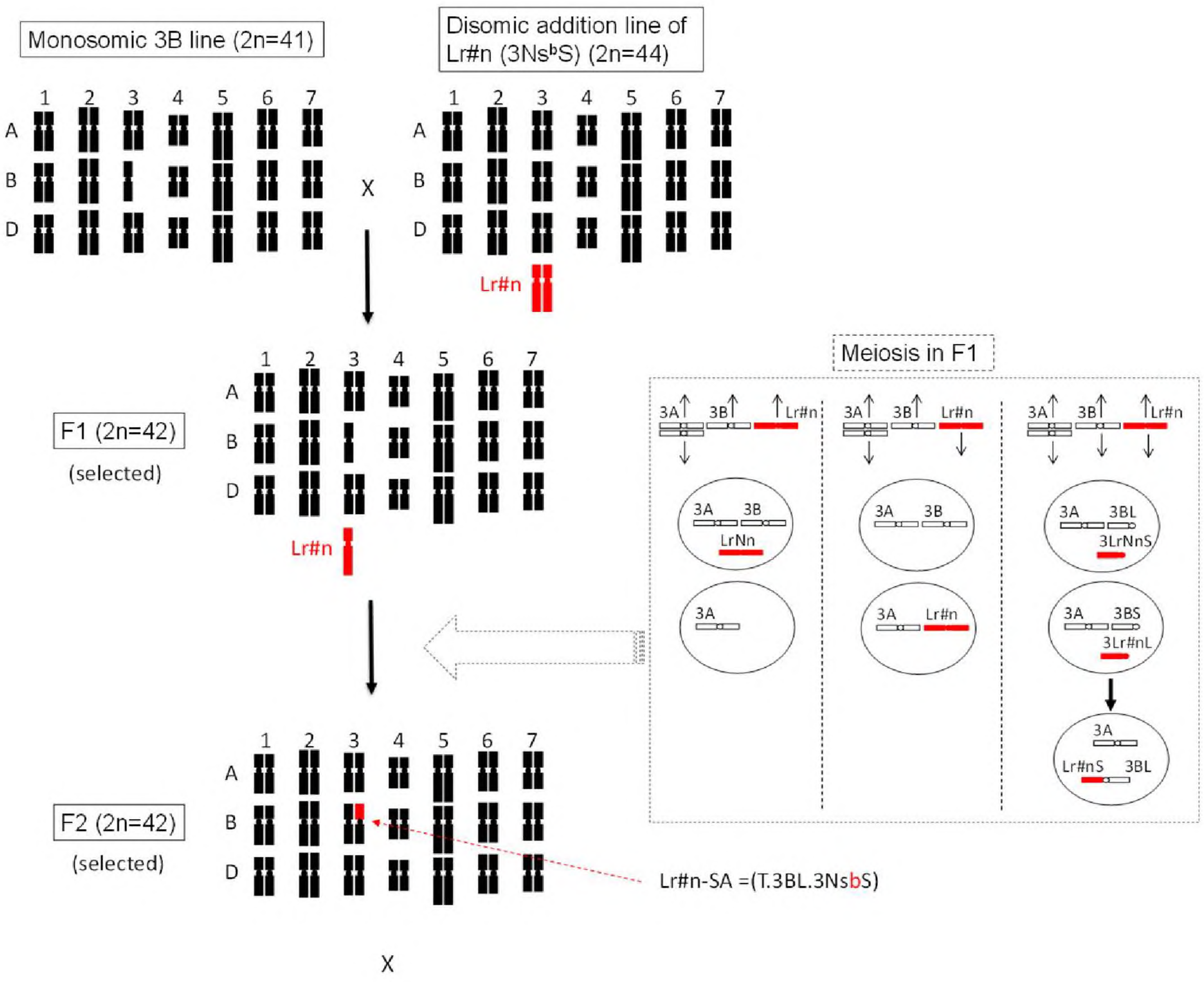

Fig. S4a. Development of centromeric translocation lines using wheat monosomic lines. The development of Lr#n-SA (T3BL.3NsbS) is shown as example.

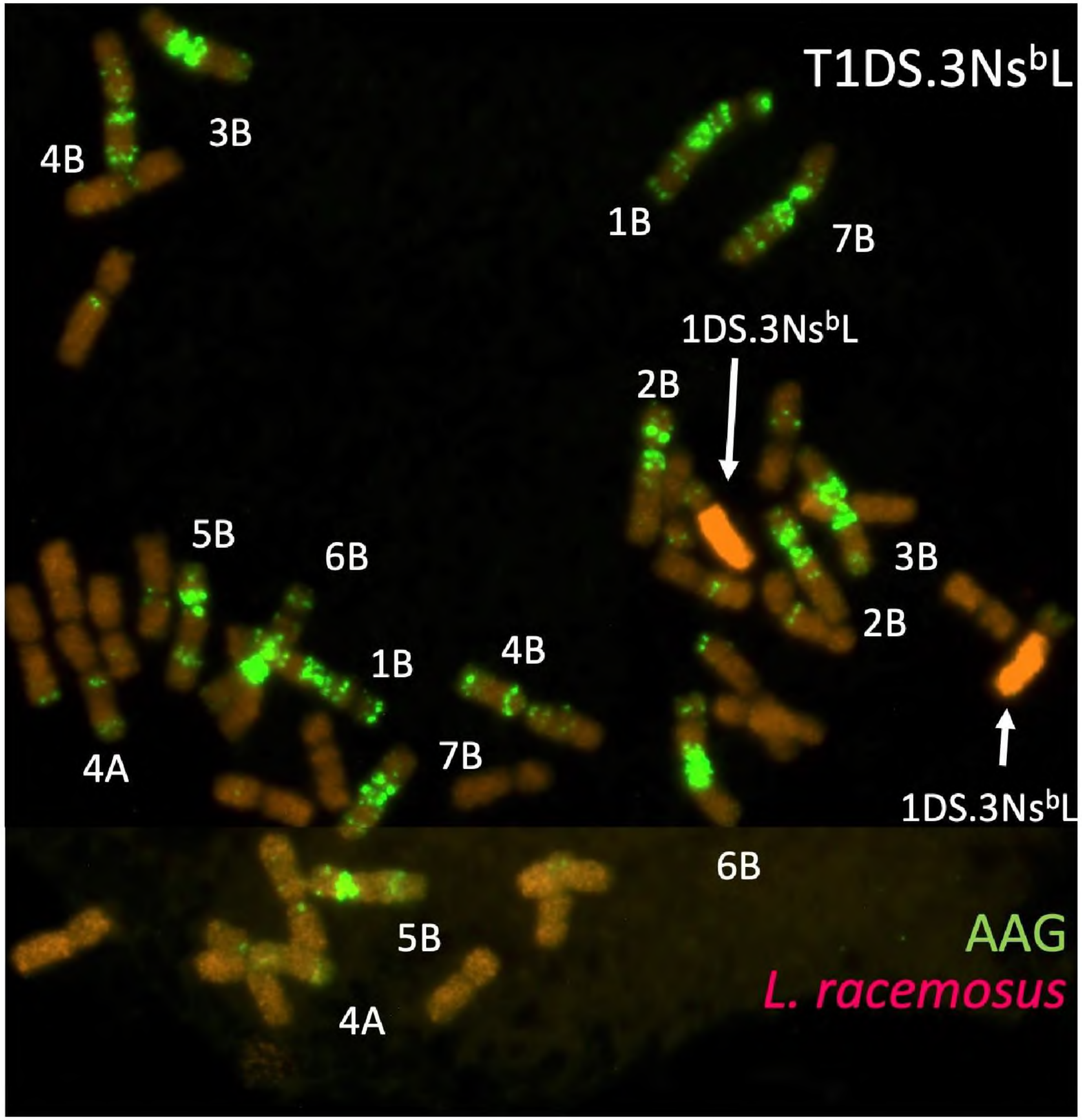

Figure S4b. Lr#n-LA complete long-arm translocation on wheat chromosome 3B in 'Chinese Spring'

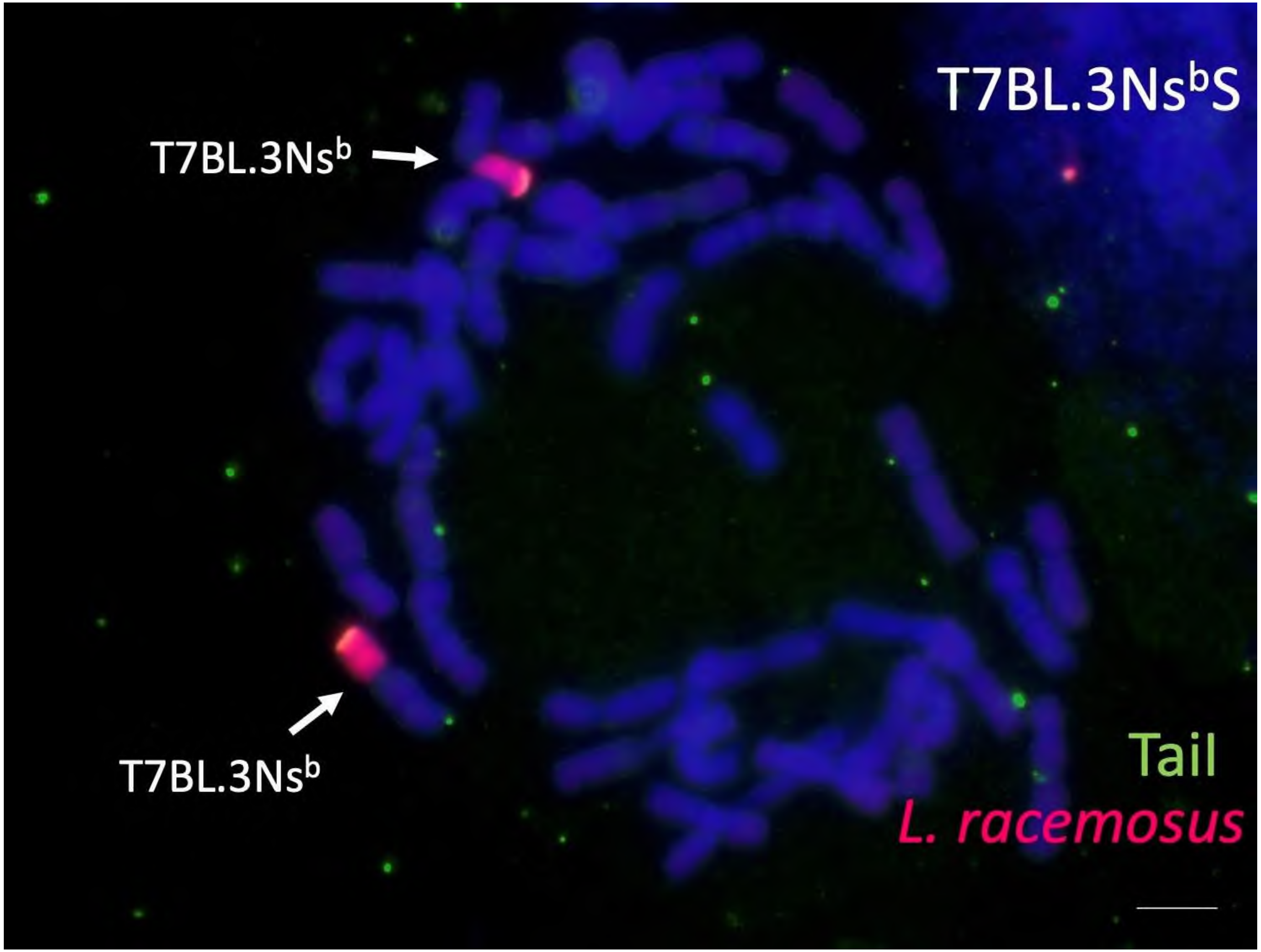

Figure S4c. Lr#n-SA complete short-arm translocation on wheat chromosome 7B translocation in 'Chinese Spring'

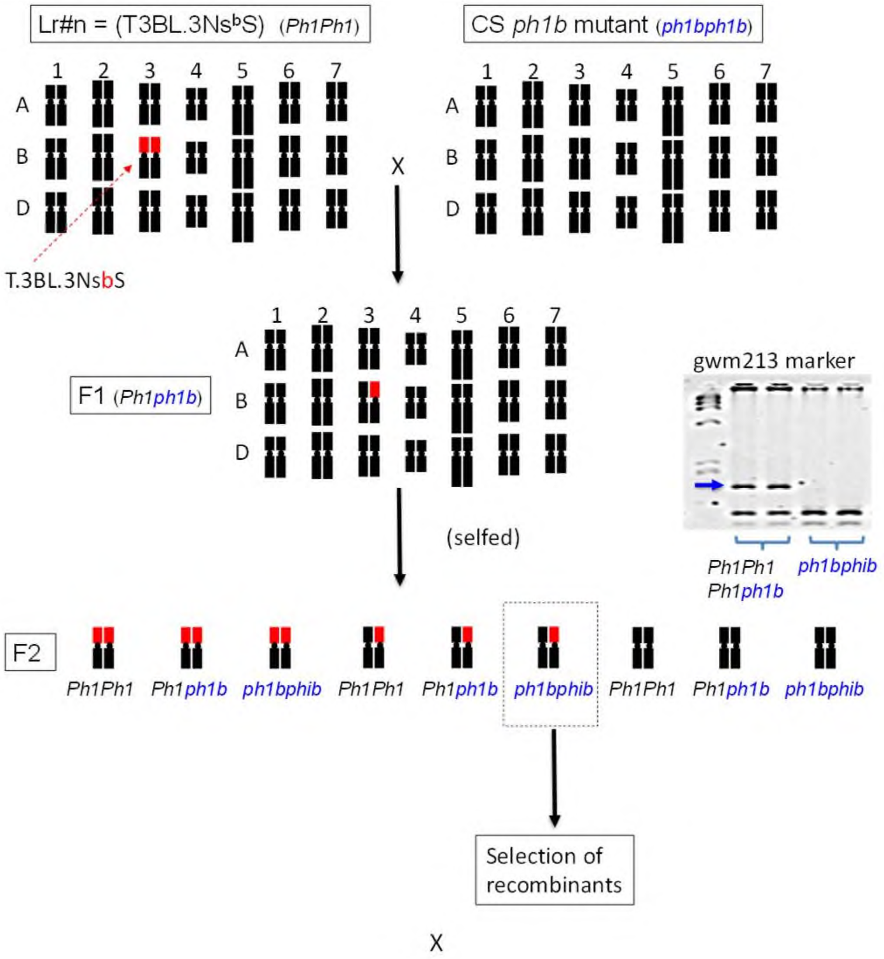

Fig. S5. Shortening of Lr#n-SA (T3BL.3Ns^{b}S) translocation lines using CS ph 1b line.
Lr#n-SA (T3BL.3Ns^{b}S) is initially crossed with CS ph1b mutant where, in F1, where plants are heterozygous in Lr#n-SA and *ph1b*. In F2, the plants that are heterozygous in Lr#n-SA and homozygous in ph1b were selected for shortening chromosome segment size of lr#n-SA. The selection in F2 was done by GISH for Lr#n-SA and gwm213 SSR marker for *ph1b*.

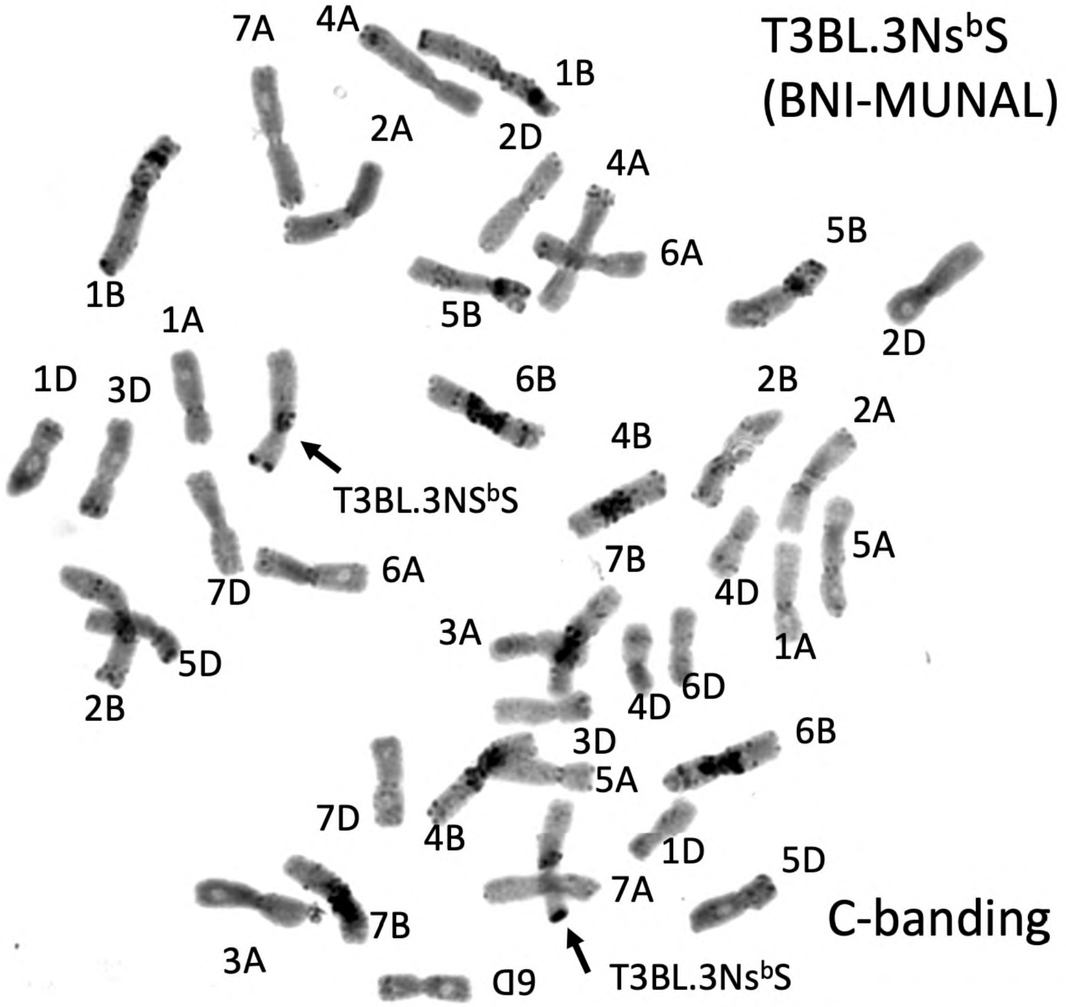

Figure S6a. Karyotype analysis (C-banding image) for 'BNI-MUNAL (T3BL.3Ns^{b}S) ' showing Lr#n-SA translocation (complete short-arm) on wheat chromosome 3B

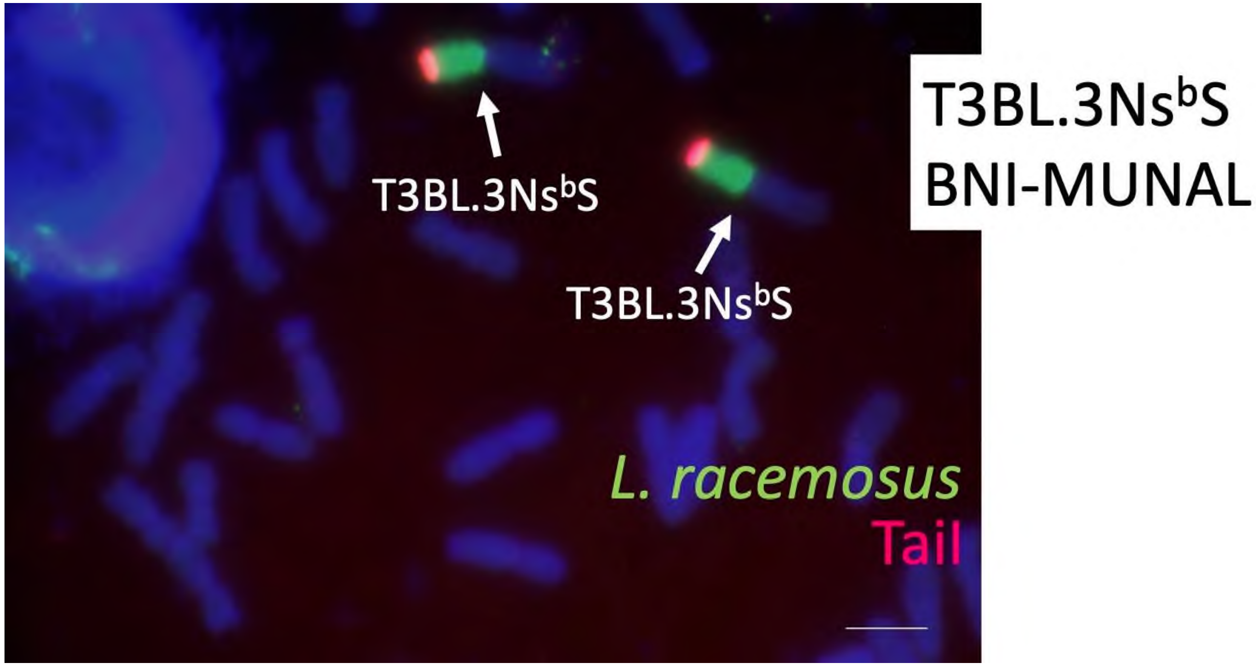

Figure S6b. Karyotype analysis for 'BNI-MUNAL (T3BL.3Ns^{b}S) ' showing Lr#n-SA translocation (complete short-arm) on wheat chromosome 3B (red: Tail family probe, green: *L. racemosus* genomic DNA)

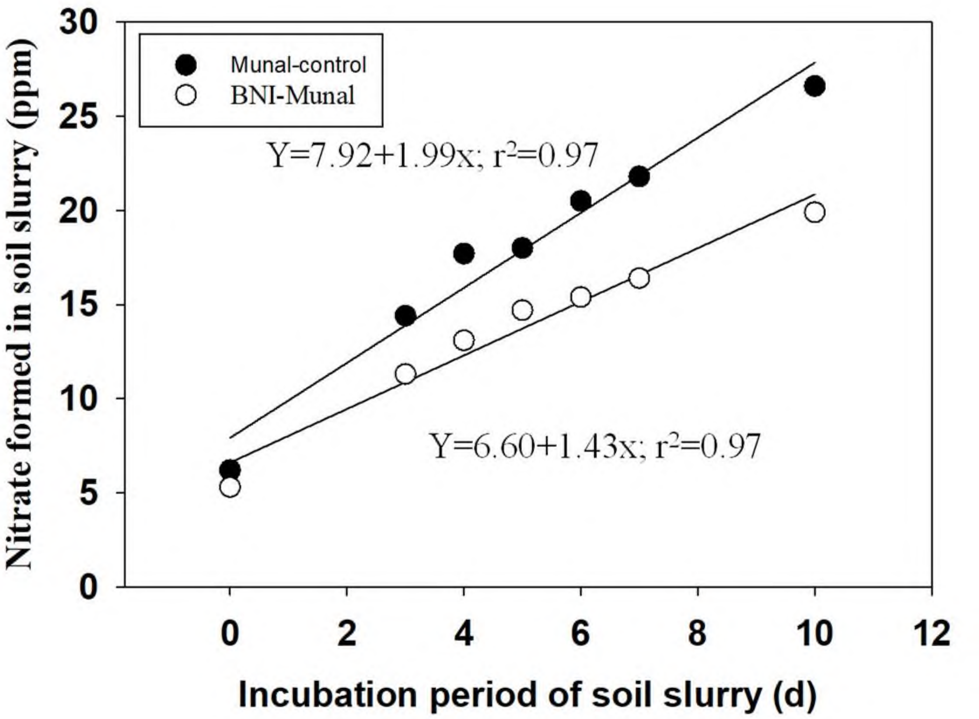

Fig. S7a. Potential nitrification in root-zone-soils collected from field grown plants of MUNAL-control and BNI-MUNAL (BNI-Isogenic lines, Field study-6a); soils were collected from 250 kg N ha^{-1} treatment field plots; Slopes of regression lines are significantly different (*P<0.001*) based on ANCOVA (analysis of covariance). Root-zone-soils were collected on 16th day after application of 2nd split of nitrogen application; Each data point represent mean of 2 replications (soils from 2 replications are pooled and considered as one replication).

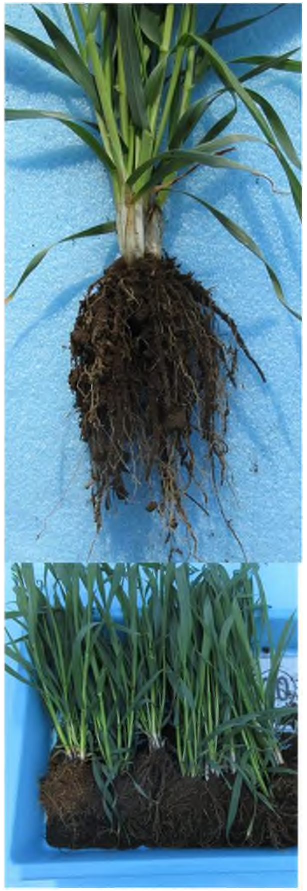

Fig. S7a1. Field grown wheat plant roots showing soil attached to roots (Field study-6a), which is considered as part of root-zone-soils, used for various soil incubation studies to characterize BNI function

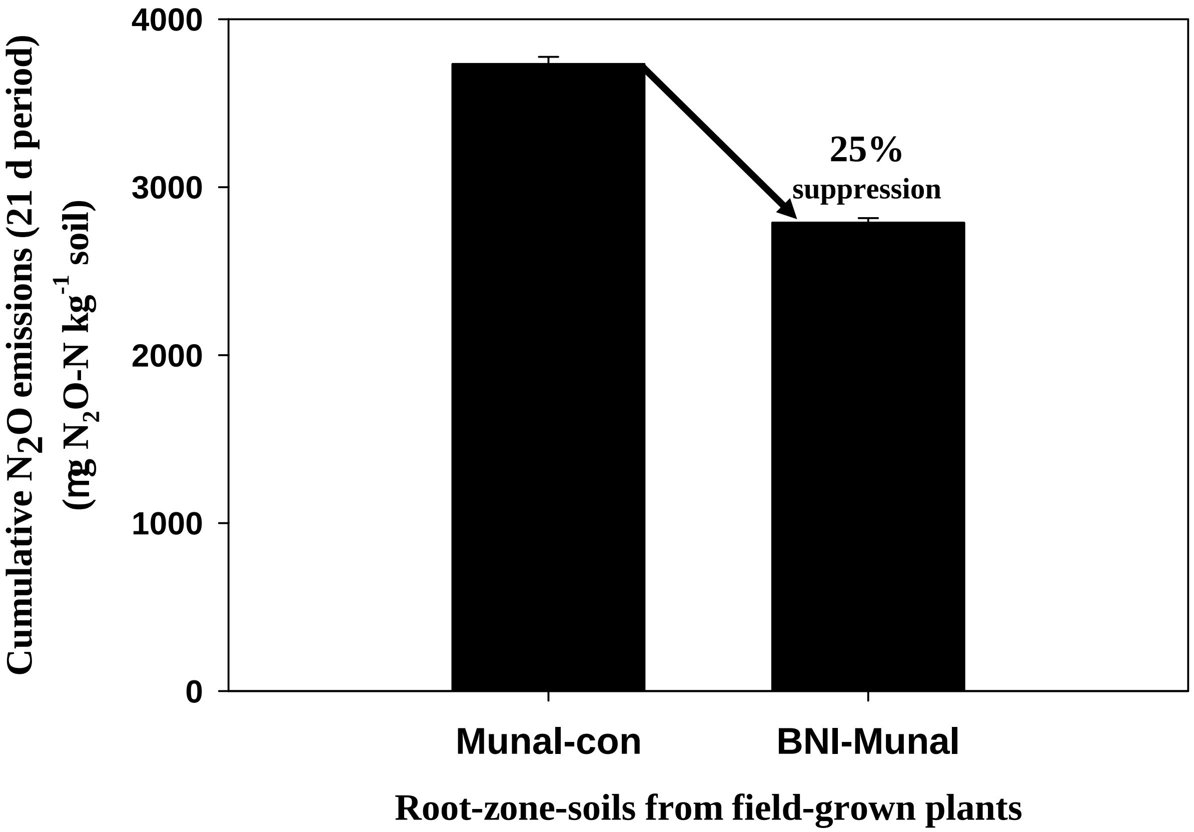

Fig. S7b. Cumulative N_2O emissions from root-zone-soils collected from MUNAL-control and BNI-MUNAL (BNI-Isogenic lines; Field study-6a). Root-zone-soils are collected on 16th day from 250 kg N ha^{-1} field-plots after application of 2nd split of nitrogen fertilizer applications. Five g of soil was incubated for 21d using 100 ml glass vial at 20 ° C with 80% relative humidity in the incubator. Soils were incubated with 250 mg N kg^{-1} soil as $(NH_4)_2SO_4$ and moisture levels were maintained at 70% WFPS during the incubation period (*Values are means* $\pm$ *SE of four replications*). N_2O emissions were monitored daily during 21 d period (also see Fig. 5b) and cumulative N_2O emissions during the incubation period are presented here. Based on two-way ANOVA using GLM model with SYSTAT 11.0; SE of LS mean = 117.8; Root-zone-soils collected from BNI-MUNAL have significantly ($P<0.01$) lower N_2O emissions than MUNAL-control.

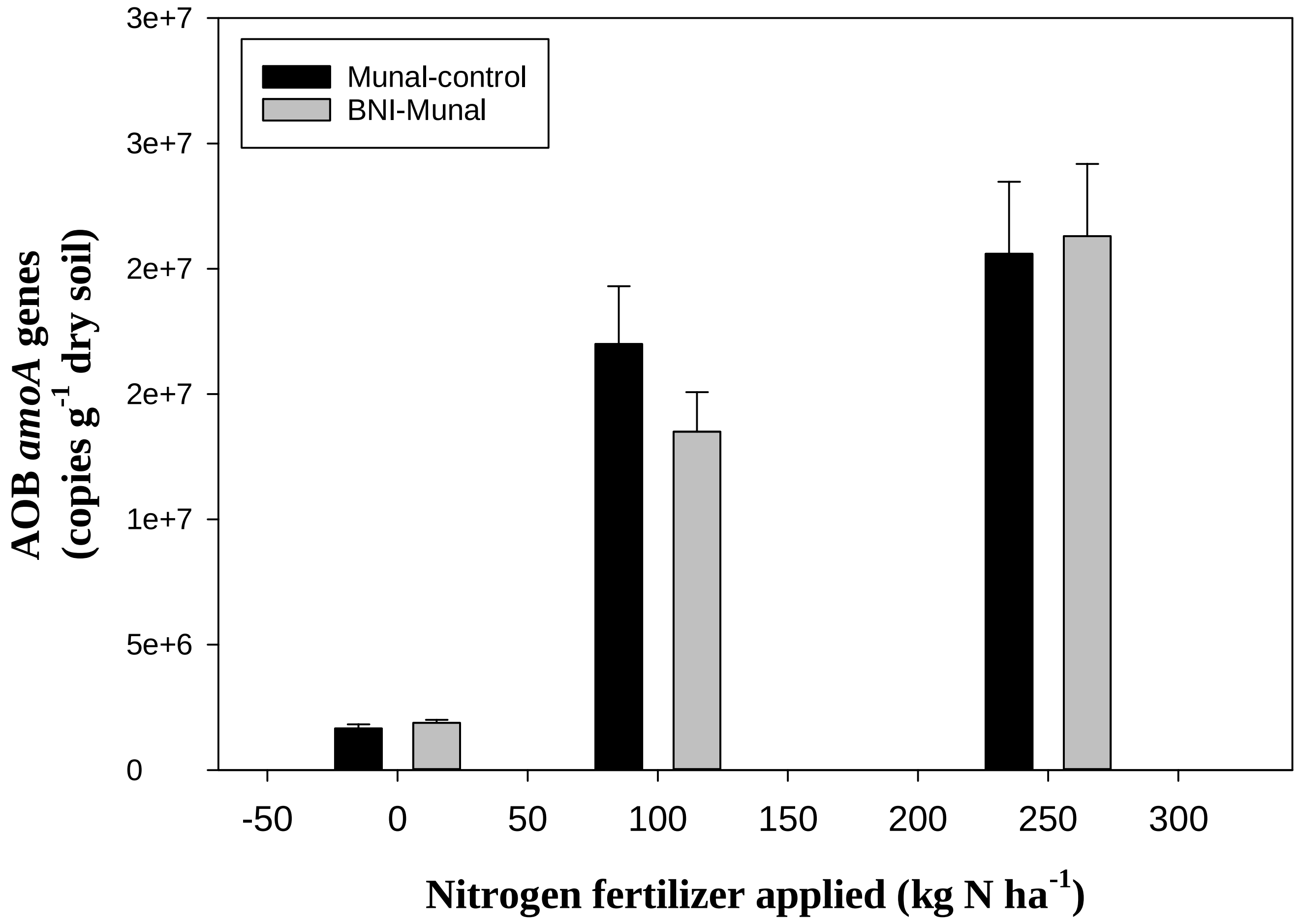

Fig. S7c. AOB populations in root-zone-soils of field grown plants of BNI-Isogenic lines (MUNAL-control *vs* BNI-MUNAL; Field study-6a, soil pH is 5.0 to 5.5). Results suggest that BNI-MUNAL suppress AOA more effectively compared to AOBs (also see Fig. 5b). Root-zone-soil samples are taken after 16th day after of 2nd split nitrogen fertilizer. Values are means $\pm$ SE of four replications (Based on three-way analysis of data using GLM model with SYSTAT 11.0; Genetic stock effect on AOB are non-significant; but N-treatment effects on AOB are significant ($P<0.001$).

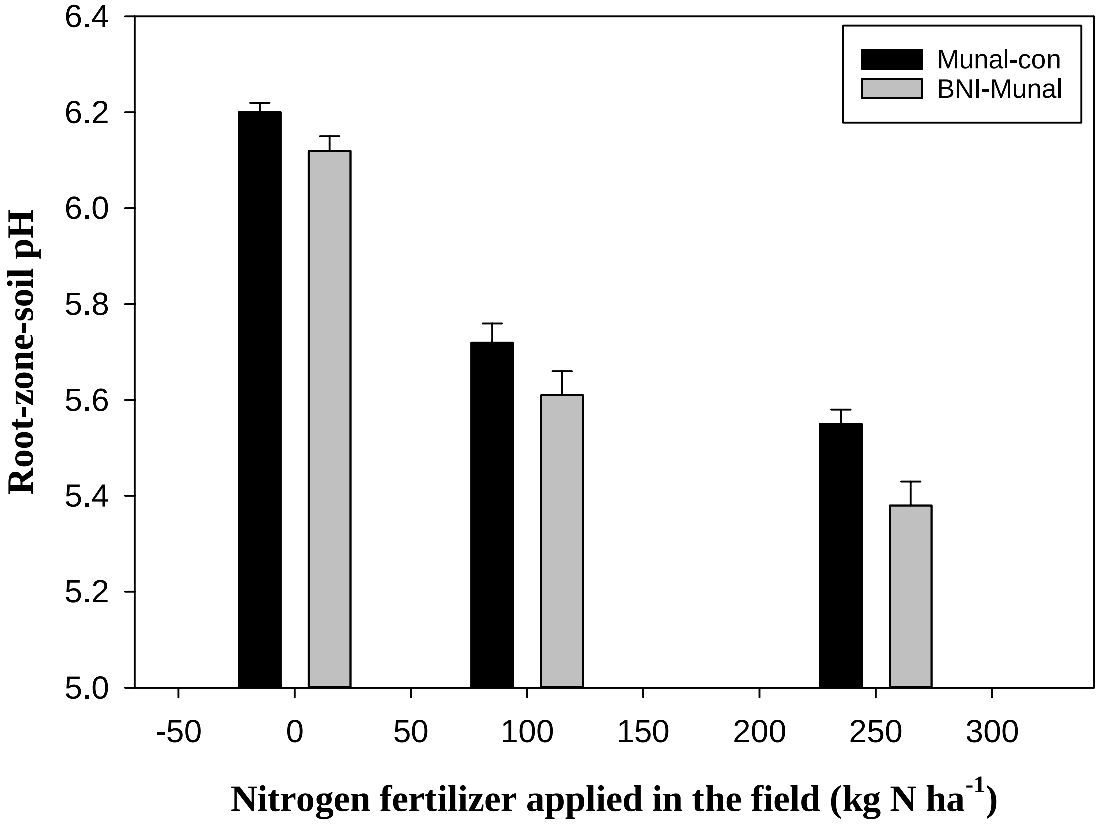

Fig. S7d. pH of the root-zone-soil of BNI-isogenic lines 'MUNAL-control and 'BNI-MUNAL' (Field study-6a). Root-zone-soil samples are taken after 16th day after of 2nd split nitrogen fertilizer . Three-way ANOVA using GLM with SYSTAT 14.0; SE of LS Mean (Genetic stock) 0.0139 (*P<0.001*); SE of LS Mean (N-Tr) = 0.017 (*P<0.001*) (*Values are means* $\pm$ *SE from four replications).*

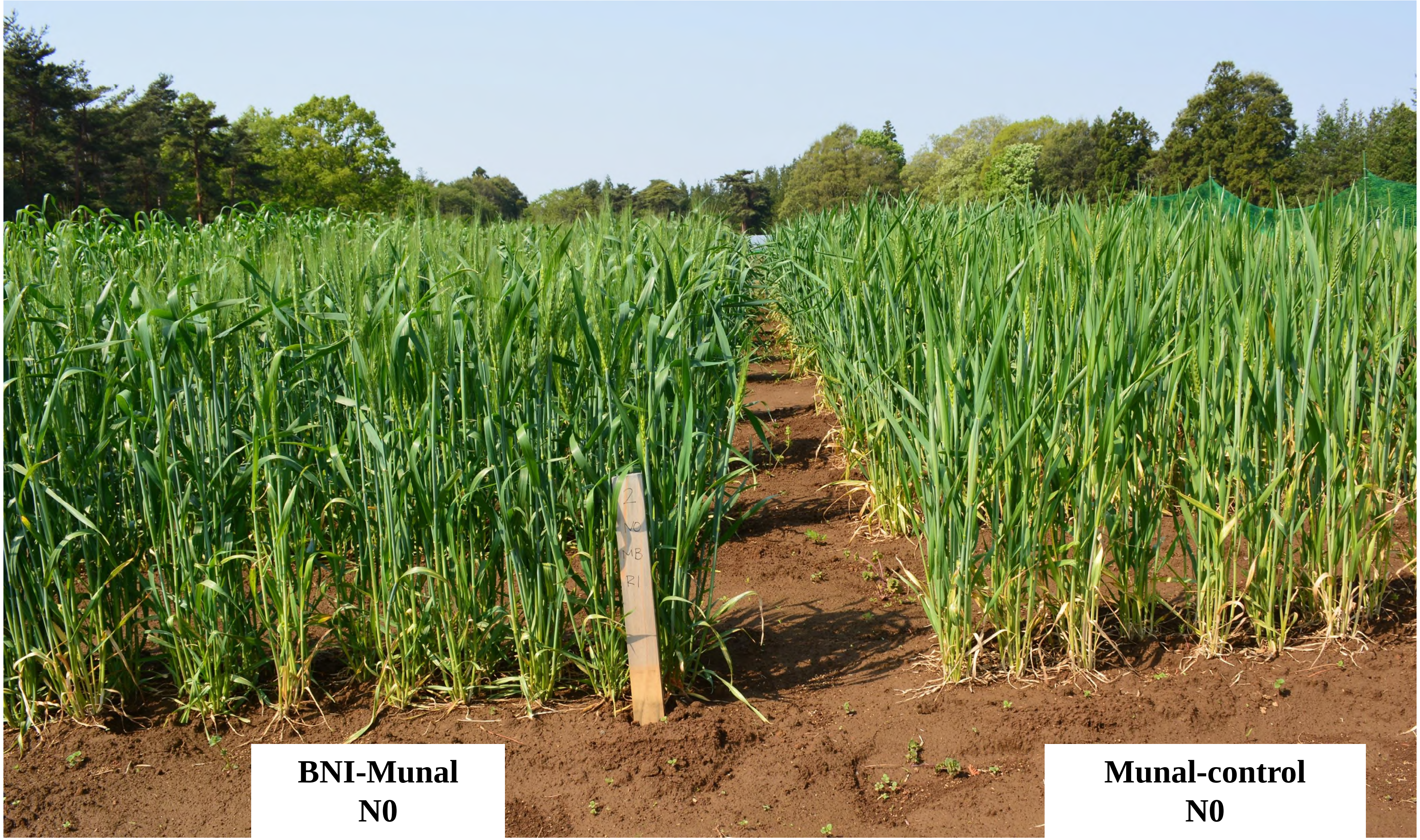

Fig. S8a. 'BNI-MUNAL' (i.e. 'MUNAL' carrying BNI-trait, Lr#n-SA) and 'MUNAL-control' (BNI-Isogenic lines; Field study-6a) at heading stage under N0 treatment in the Hachimantai JIRCAS field, Tsukuba, Japan. Mineralized soil-nitrogen levels are about 5.0 ppm at the time of planting. Nitrogen deficiency symptoms (i.e. pale-yellow leaves) have appeared in 'MUNAL-control', but not in 'BNI-MUNAL', which remained healthy green till physiological maturity.

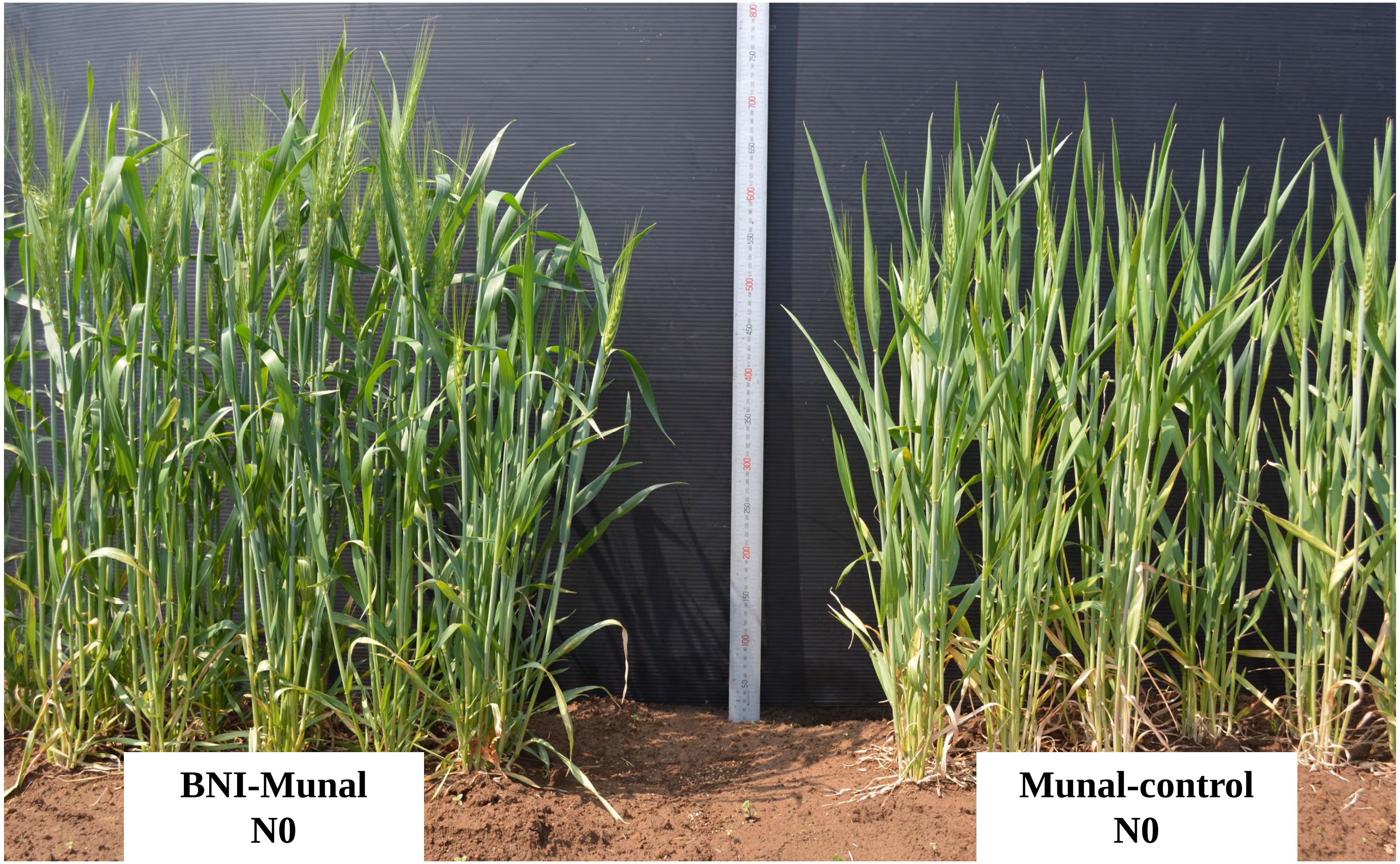

Fig. S8b. 'BNI-MUNAL' (i.e. MUNAL cv carrying BNI-trait, Lr#n-SA) and 'MUNAL-control' (BNI-Isogenic lines; Field study-6a) at heading stage under N0 treatment in the Hachimantai JIRCAS field, Tsukuba, Japan. Mineralized soil-nitrogen levels are about 5.0 ppm at the time of planting. Nitrogen deficiency symptoms (i.e. pale-yellow leaves) have appeared in 'MUNAL-control', but not in 'BNI-MUNAL', which remained healthy green till physiological maturity. Black sheet was placed behind to have a good contrast to see visually the nitrogen deficiency symptoms in 'MUNAL-control' for the photograph purpose.

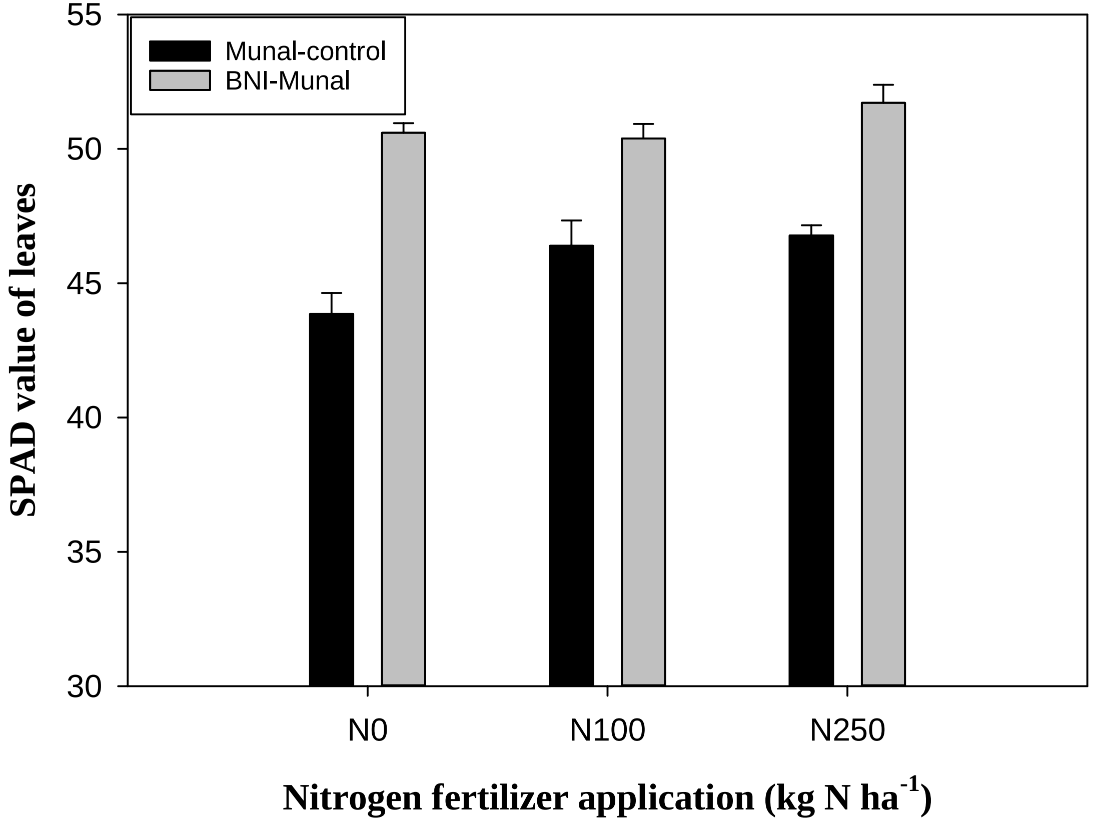

Fig. S8c. SPAD values (an indication for chlorophyll levels in leaves) measured for field grown wheat genetic stocks 'MUNAL-control' and 'BNI-MUNAL' (BNI-Isogenic lines; Field study-6a). SPAD measurements taken 7d after 3rd split nitrogen fertilizer application. Wheat plants were at heading stage at the time of measurements Three-way ANOVA using GLM with SYSTAT 14.0; SE of LS Mean (Genetic stock) 0.42 (*P<0.001*); SE of LS Mean (N-Tr) 0.51 (*P<0.05*) (*Values are means* ± *SE from four replications).*

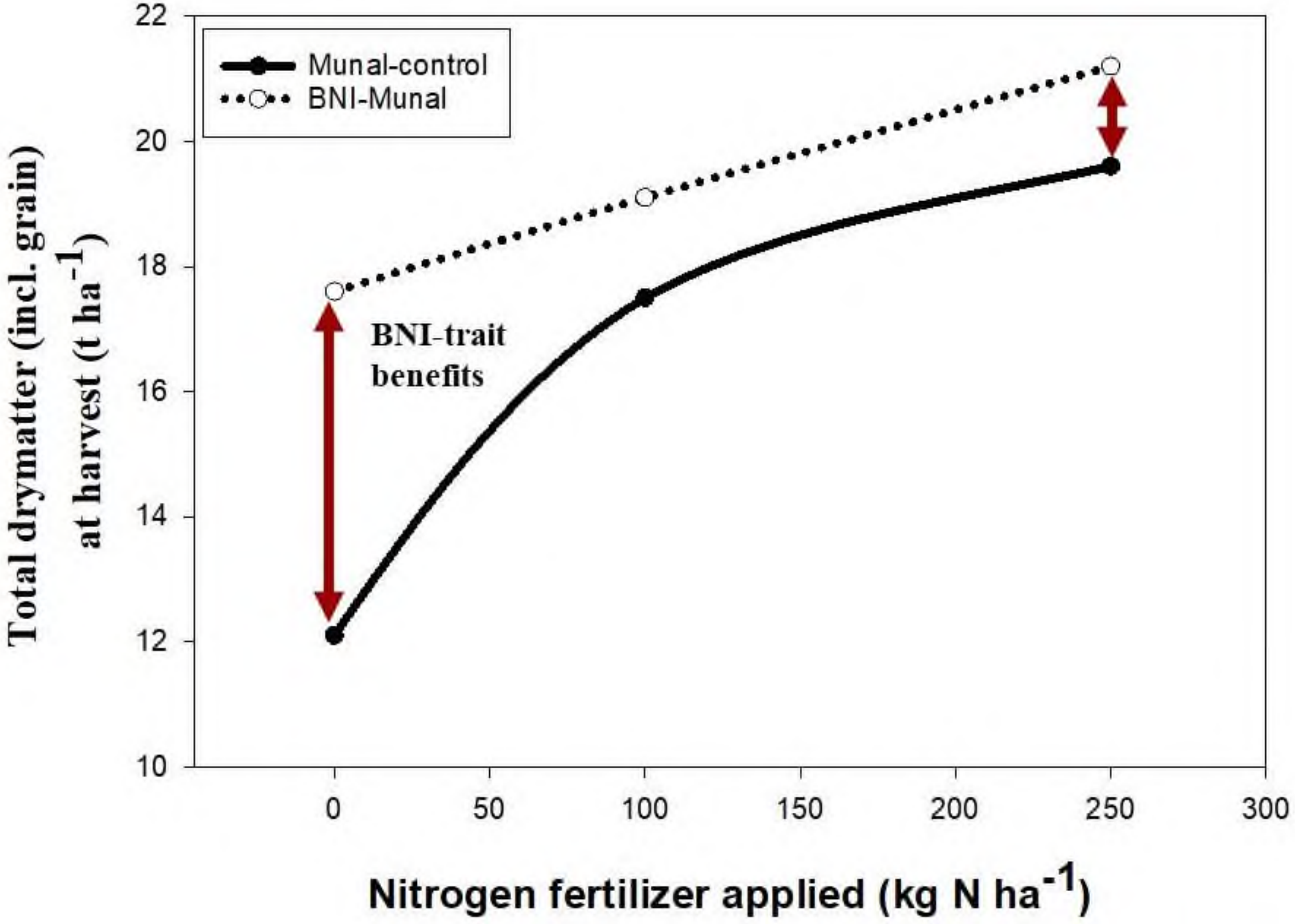

Fig. S8d. Total dry matter produced (including grain) in wheat genetic stocks 'MUNAL-control' vs. 'BNI-Munal' (BNI-Isogenic lines; Field study-6a) under various nitrogen fertilizer treatments. Detailed statistical analysis on results on total dry matter produced, agronomic traits and yield components are presented in Table S7.

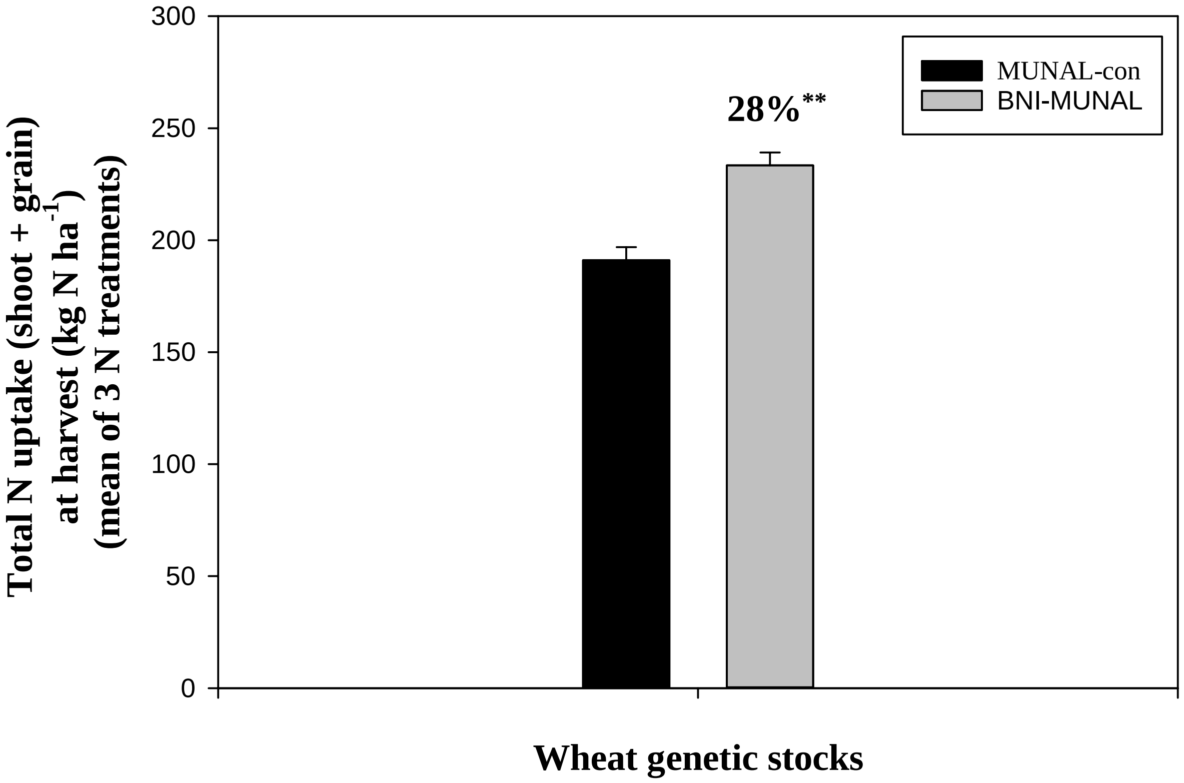

Fig. S9a . Total N uptake at harvest (above ground dry matter that include both shoot and grain; mean of all three N-treatments for wheat genetic stocks 'MUNAL-control' vs. 'BNI-MUNAL') (BNI-Isogenic lines; Field study-6a). Three-way ANOVA using GLM with SYSTAT 14.0; SE of LS Mean (Genetic stock) 5.79 (*P<0.001*); (*Values are means* $\pm$ *SE from four replications)*

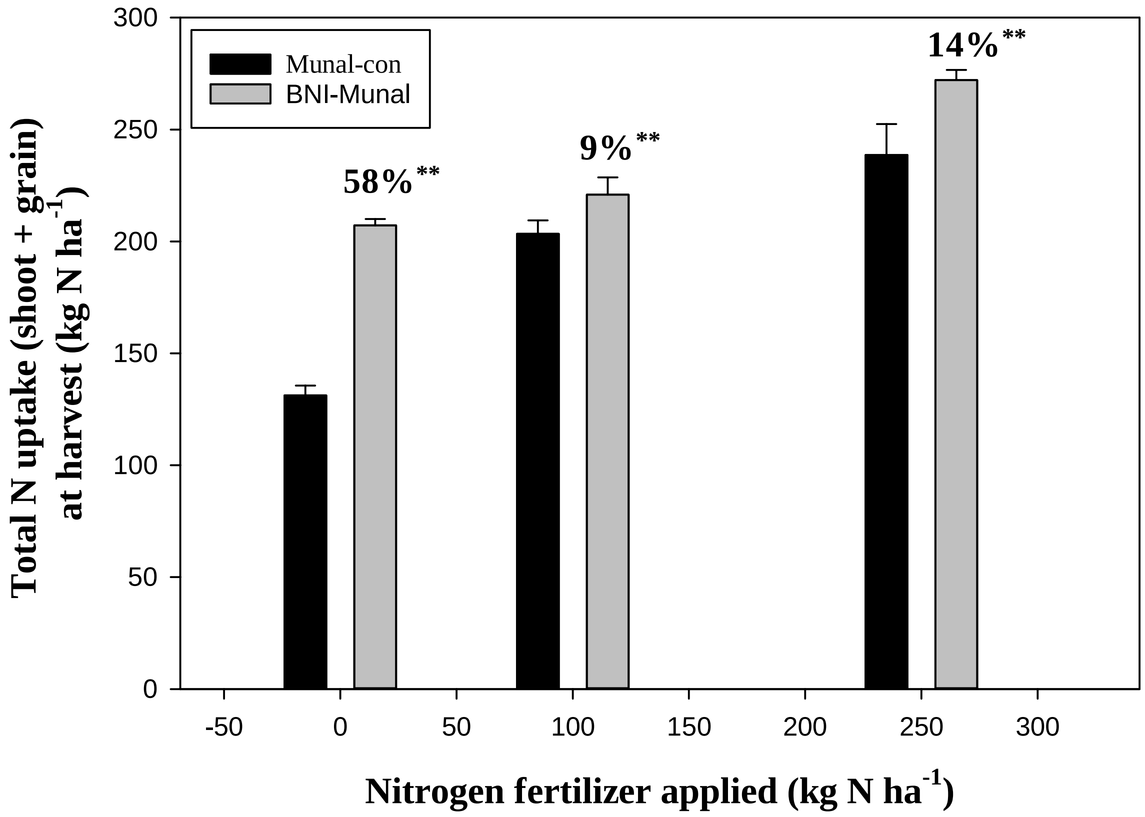

Fig. S9b . Total N uptake at harvest (above ground dry matter that include both shoot and grain) in wheat genetic stocks 'MUNAL-control' vs. 'BNI-MUNAL' (BNI-Isogenic lines; Field study-6a). Three-way ANOVA using GLM with SYSTAT 14.0; SE of LS Mean (Genetic stock) 5.79 (*P<0.001*); SE of LS Mean (N-Tr) 7.10 (*P<0.001*) (*Values are means* ± *SE from four replications).*

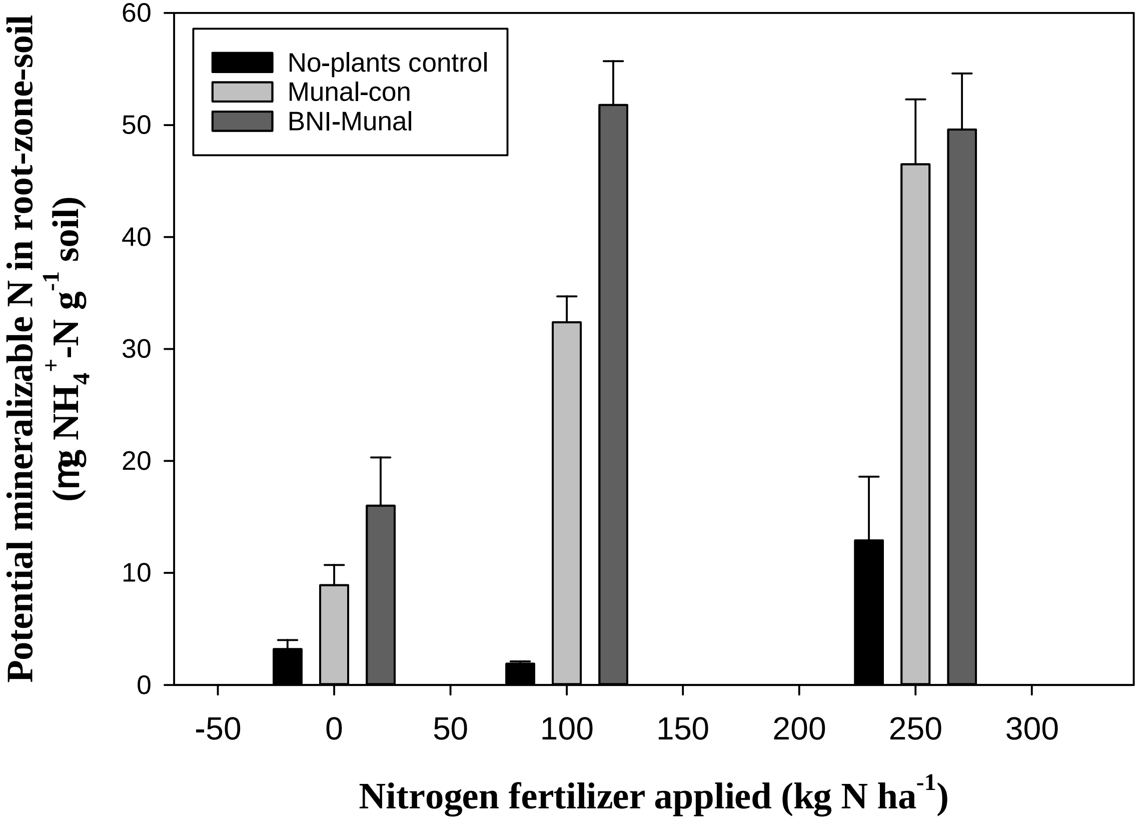

Fig. S9c. Potential mineralizable N in root-zone-soils (after 7d incubation period) of MUNAL-control and BNI-MUNAL (BNI-Isogenic lines; Field study-6a). The results suggest that BNI-MUNAL has a stronger influence on SOM mineralization compared to MUNAL-control. Root-zone-soil samples were taken on 16th day after 2nd split Nitrogen fertilizer application to each experimental plot in the field. Three-way ANOVA using GLM with SYSTAT 14.0; SE of LS Mean (Genetic stock) 3.18 (*P<0.05*); SE of LS Mean (N-Tr) 3.89 (*P<0.001*) (*Values are means* $\pm$ *SE of four replications).*

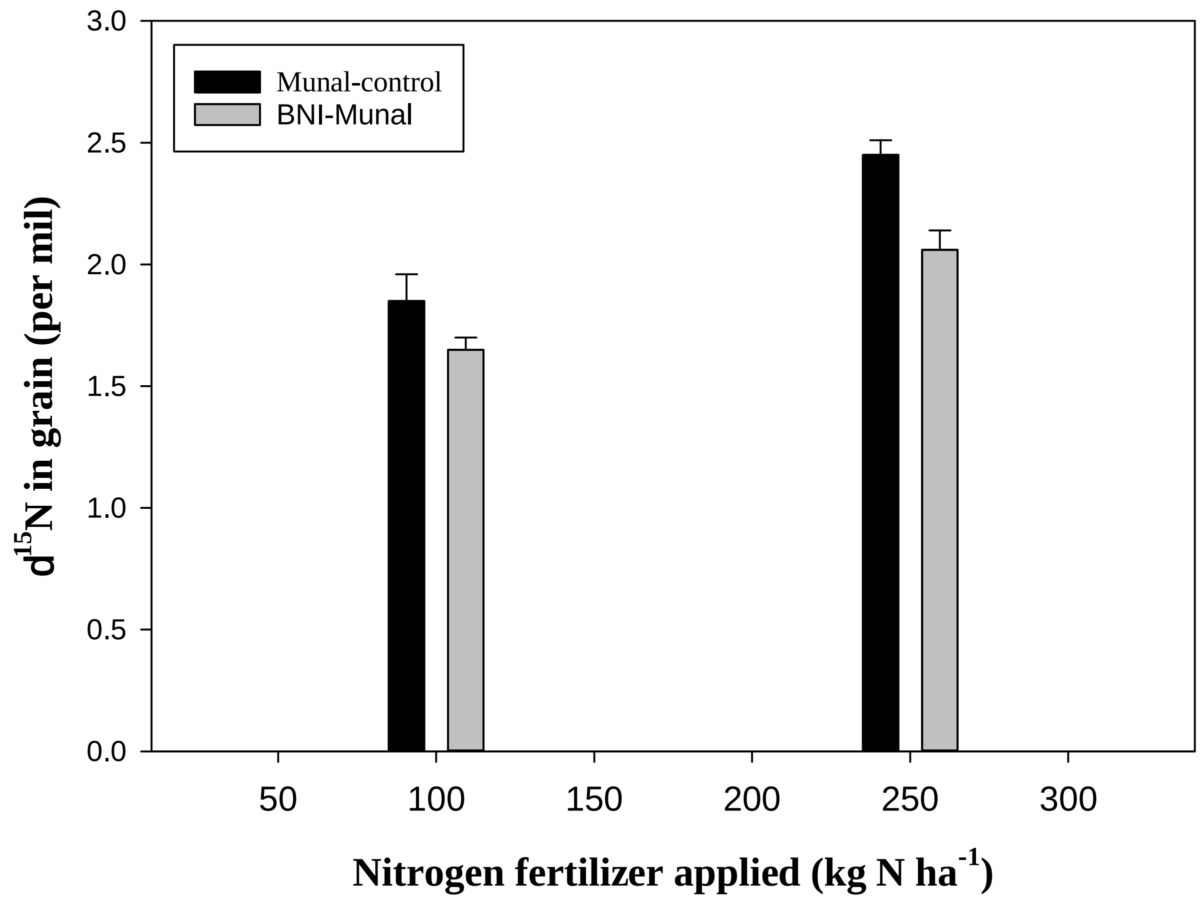

Fig. S9d . Grain d^{15}N levels in 'MUNAL-control' vs 'BNI-MUNAL' (BNI-Isogenic lines; Field study-6a). A lower grain d^{15}N in 'BNI-MUNAL' is an indication for higher NH_4^+ assimilation compared to 'MUNAL-control' during the growth period Three-way ANOVA using GLM with SYSTAT 14.0; SE of LS Mean (Genetic stock) 0.05 (*P<0.002*); SE of LS Mean (N-Tr) 0.05 (*P<0.001*) (*Values are means ± SE of four replications)*. About 16% lower d^{15}N in grain suggests the possibility of 16% more ammonium uptake and assimilation in BNI-MUNAL compared to MUNAL-control with 250 kg N ha^{-1} field plots.

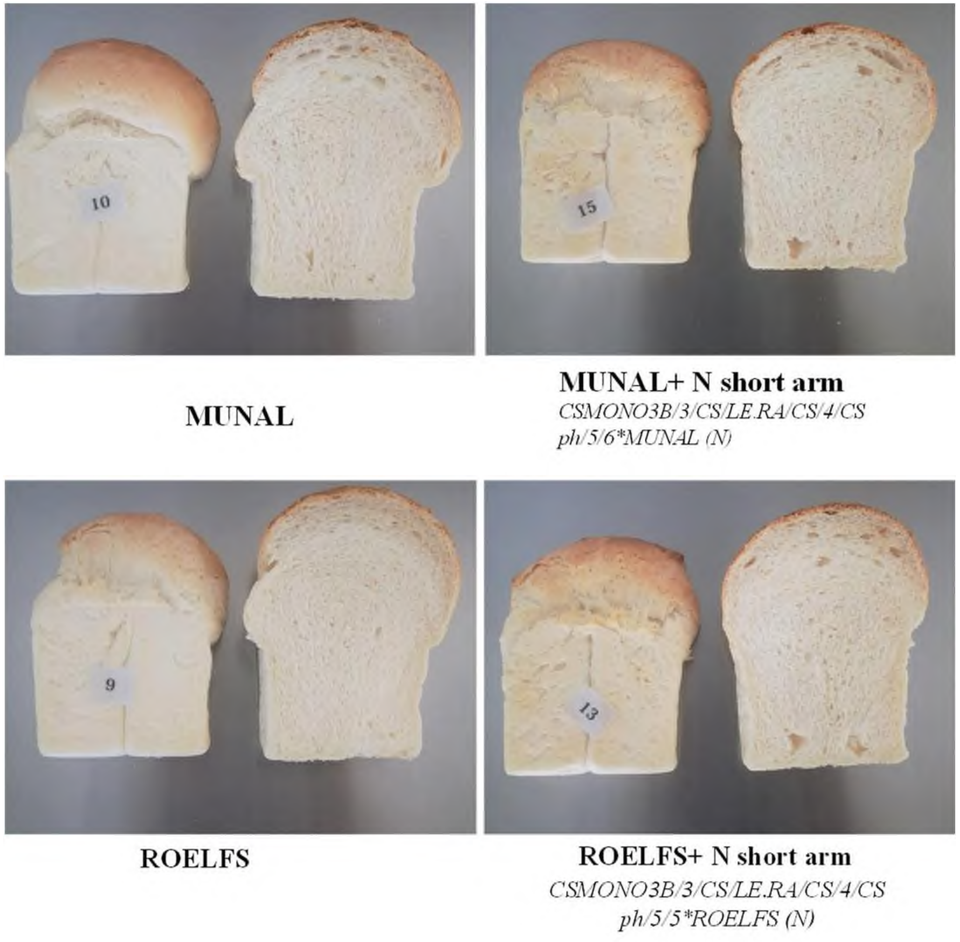

Fig. S10. Bread characteristics of 'MUNAL-control vs. BNI-MUNAL; ROELFS-control vs BNI-ROELFS (BNI-Isogenic lines; grain used for bread making testing came from field studies in Mexico Study 6c and 6d).

Table S1. BNI-capacity of CIMMYT-derived wheat varieties released from 1950 to 2010 period (Study-1). Data is subjected to GLM ANOVA using SYSTAT 14.0 (*Values are means* + *SE of four replications*).

Serial no.	Wheat variety	**BNI activity released from roots (ATU g^{-1} root dry wt. d^{-1})**	
		Mean	*SE*
1	Yaqui 50	54.0	*3.8*
2	Penjamo 62	33.2	*10.2*
**3**	**Sonora 64**	**89.1**	***10.3***
4	Inia F 66	56.2	*11.0*
5	Siete Cerros T 66	63.7	*8.1*
6	Jupateco F 73	51.8	*9.1*
7	Pavon F 76	8.3	*3.7*
8	Genaro T 81	24.6	*11.4*
9	Seri M 82	18.1	*7.4*
10	Bacanora T 88	40.0	*14.4*
11	Rayon F 89	27.1	*10.7*
12	Baviacora M 92	23.4	*15.6*
13	Villa Juarez F 2009	61.7	*8.7*
**14**	**Roelfs F 2007**	**88.8**	***9.2***
15	Borlaug M 95	59.4	*13.7*
16	Tacupeto F 2001	44.6	*12.4*
17	Kronstad F 2004	65.8	*4.8*
18	Navojoa M 2007	77.4	*4.8*
19	Norin 10	42.9	*9.9*
**20**	**Chinese Spring**	**72.3**	***7.7***
	SEM	*8.8***	

Table S2a. The list of wild species and wheat-wild species complete or partial amphiploid lines used in this study.

Serial No.	Line	Wheat parent	Genome	Alien parent	Genome	Accession number of alien plant	Number of chromosomes
1	*Leymus racemosus*				**NsNsXmXm or** $Ns^1Ns^1Ns^2Ns^2$	**MK0001**	**2n = 28**
2	***T. tur/E.mollis***	*Triticum turgidum*	**AABB**	*Leymus mollis*	**NsNsXmXm or** $Ns^1Ns^1Ns^2Ns^2$	**Unknown**[*2]	**2n = 40**
3	**DVERD-S/PS.JU//68112/WARD**	*T. turgidum ssp. durum*	**AABB**	*Psathyrostachys juncea*	**NsNs**	**PI531828**	**2n = 42**
4	**CS/TH.BESSRABICUM**	*T. aestivum*	**AABBDD**	*Thinopyrum bessarabicum*	**JJ**		**2n = 56**
5	**CS/TH.BESSRABICUM**[*1]	*T. aestivum*	**AABBDD**	*Thinopyrum bessarabicum*	**JJ**	**PI531712**	**2n = 56**
6	**CS/TH.ELONGATUM**	*T. aestivum*	**AABBDD**	*Thinopyrum elongatum*	**EE**	**CS-5-71**	**2n = 56**
7	**CS/TH.JUNCEFORME 4X**	*T. aestivum*	**AABBDD**	*Thinopyrum junceiforme*	$J^bJ^bJ^eJ^e$	**PI414667**	**2n = 52**
8	**CS/TH.SCIRPEUM**[*1]	*T. aestivum*	**AABBDD**	*Thinopyrum scirpeum*	$J^eJ^eJ^eJ^e$	**CS-5-51**	**2n = 58**
9	**CS/TH.INTERMEDUM**[*1]	*T. aestivum*	**AABBDD**	*Thinopyrum intermedium*	$StStJ^eJ^eVV$ **or** $StStJ^rJ^rJ^{vs}J^{vs}$	**RS-10-31**	**2n = 74**
10	**CS/TH.TRICHOPHORUM**[*1]	*T. aestivum*	**AABBDD**	*Thinopyrum intermedium ssp. trichophorum*	**StStJJVV**	**RS-6-a**	**2n = 62**

Note: All amphiploid lines were developed in CIMMYT from 1980-2000 with help of Dr. Sears
**1 = obtained from dr. Merker Arnulf (Swedish University of Agricultural Sciences) in 1980's*
United States Department of Agriculture (USDA)

Table S2b. The list of wild species and wheat-wild species complete or partial amphiploid lines used in this study.

Serial no.	Line	Wheat parent (genome)	Alien parent	KSU code name	Seed source
11	**CS/A. elongatum**	*T. aestivum*	*Agropyron elongatum*	TA3425	KSU, USA
12	**Vilmorin27/A.intermedium**	*T. aestivum*	*Agropyron intermedium*	TA3646	KSU, USA
13	**Zhong7/A. intermedium**	*T. aestivum*	*Agropyron intermedium*	TA8032	KSU, USA
14	**Summer1/A. intermedium**	*T. aestivum*	*Agropyron intermedium*	TA8036	KSU, USA
15	**OK7211542/A.intermedium**	*T. aestivum*	*Agropyron intermedium*	TA8038	KSU, USA
16	**CS/A. scirpeum**	*T. aestivum*	*Agropyron scirpeum*	TA3426	KSU, USA
17	**T.timopheevii subsp. Timopheevii/C.bogdanii**	*Triticum timopheevii subsp. Timopheevii*	*Critesion bogdanii*	TA3412	KSU, USA
18	**CS/C. californicum**	*T. aestivum*	*Critesion californicum*	TA3443	KSU, USA
19	**Inayamakomugi/E.cillaris**	*T. aestivum*	*Elymus ciliaris*	TA3427	KSU,USA
20	**T. aestivum/A.distichum**	*T. aestivum*	*Agropyron distichum*	TA3409	KSU, USA

Table S2c. BNI-capacity of amphiploid genetic stocks of wild-wheat species and wild-grasses (**Study-2b**). (*Values are means + SE from of replications*).

**Serial No.**	**Amphiploid genetic stock**	**BNI activity released from roots (ATU g^{-1} root dry wt. d^{-1})**	
		**Mean**	*SE*
1	*Psathyrostachys juncea*	63.1	*17.9*
2	*Thinopyrum bessarabicum-1*	0.4	*8.2*
3	*Th. bessarabicum-2*	44.3	*13.3*
4	*Th. elongatum*	95.8	*16.9*
5	*Th. junceiforme*	20.6	*5.6*
6	*Th. scirpeum*	24.8	*12.1*
7	*Th. intermedium*	Negative activity	
8	*Th. trichophorum*	Negative activity	
9	*Agropyron elongatum* (TA3425)	Negative activity	
10	*A. intermedium* (TA3646)	Negative activity	
11	*A. intermedium* (TA8032)	Negative activity	
12	A. *intermedium* (TA8036)	Negative activity	
13	*A. intermedium* (TA8038)	Negative activity	
14	*A. scipeum* (TA3426)	Negative activity	
15	*B. bogdanii* (TA3412)	Negative activity	
16	*Critesion californicum (TA3443)*	Negative activity	
17	*Elymus ciliaris* (TA3427)	Negative activity	
18	*A.* Distichum (TA3409)	Negative activity	
**19**	***Leymus mollis*** *(T. turgidum x L. mollis)*	**643.9**	*89.7*
**20**	**Chinese Spring**	**72.8**	*15.0*

Note: Lines #1-6 are corresponding to #3-10 in Table S2a, and line #19 is to #2.

Table S2d. The list of bread wheat and alien chromosome addition/translocation lines used in this study

Serial No.	Variety/Genetic stock	Pedigree	Reference web-site for pedigree or description
1	MUNAL-1 (= SUPER-172)	WAXWING*2/KIRITATI	http://wheatpedigree.net/sort/show/92566
2	ROELFS-F-2007	KAMBARA-1*2/KUKUNA	http://wheatpedigree.net/sort/show/110286
3	QUAIU-1	BABAX/Lr42//BABAX*2/VIVITSI	http://wheatpedigree.net/sort/show/107504
4	VOROBEY	CROC-1/AE.SQUARROSA (224)//OPATA-M-85/3/PASTOR	http://wheatpedigree.net/sort/show/96759
5	NAVOJOA-M-2007	ATTILA/PASTOR	http://wheatpedigree.net/sort/show/110285
6	BORLAUG-100 (REEDLING-1)		http://wheatpedigree.net/sort/show/116519
7	SONORA-64	YAKTANA-54//NORIN-10/BREVOR/3/2*YAQUI-54	http://wheatpedigree.net/sort/show/58265
8	Lr#n	CS*2/LE.RA	*Leymus racemosus* N chromosome (Lr#n = $3Ns^b$) disomic addition line
9	Lr#n-SA	CSMONO3B/3/CS*2/LE.RA	Centromeric translocation of Lr#n short arm with wheat 3B chromosome long arm ($T3BL.3Ns^bS$)
10	Lr#n-SA-7B	CSMONO7B/3/CS*2/LE.RA	Centromeric translocation of Lr#n short arm with wheat 7B chromosome long arm ($T7BL.3Ns^bS$)
11	Lr#n-LA	CSMONO3B/3/CS*2/LE.RA	Centromeric translocation of Lr#n long arm with wheat 3B chromosome short arm ($T3SL.3Ns^bL$)
12	Tr#3	CS MONO 3B/3/CS/LE.RA//CS	Recombinant of Lr#n
13	Tr#4	CS MONO 3B/3/CS/LE.RA//CS/4/CS ph (N)	Recombinant of Lr#n
14	Tr#7	CS MONO 3B/3/CS/LE.RA//CS/4/CS ph (N)	Recombinant of Lr#n
15	BNI-MUNAL	CSMONO3B/3/CS/LE.RA//CS/4/CS ph/5/6*MUNAL (N)	Lr#n-SA in MUNAL background (BC_5F_4)
16	BNI-ROELFS	CSMONO3B/3/CS*2/LE.RA/3/CS ph/4/5*ROELFS (N)	Lr#n-SA in ROELFS background (BC_4F_4)
17	BNI-ROELFS (BC_6)	CSMONO3B/3/CS*2/LE.RA/3/CS ph/4/7*ROELFS (N)	Lr#n-SA in ROELFS background (BC_6F_3)
18	BNI-QUAIU	CSMONO3B/3/CS/LE.RA//CS/4/5*QUIAU(N)	Lr#n-SA in QUAIU background (BC_4F_4)
19	BNI-VOROBEY	CSMONO3B/3/CS/LE.RA//CS/4/5*VOROBEY(N)	Lr#n-SA in VOROBEY background (BC_4F_4)
20	BNI-NAVOJOA	CSMONO3B/3/CS/LE.RA//CS/4/5*NAVOJOA(N)	Lr#n-SA in NAVOJOA background (BC_4F_4)
BC = back cross generation			

Table S3. Yield performance of five CIMMYT wheat lines across global locations

International nursery name	31st ESWYT	28th SAWSN	32nd ESWYT	29th SAWSN	33rd ESWYT	30th SAWSN	34th ESWYT	47th IBWSN	48th IBWSN	49th IBWSN
Established year	**2010**	**2010**	**2011**	**2011**	**2012**	**2012**	**2013**	**2014**	**2015**	**2016**
**Variety name**	**Maximum grain yield ($t\ ha^{-1}$)**									
**MUNAL-1 (SUPER-172)**	**13.6**		**10.7**		**8.8**		**9.5**	**10.0**	**10.9**	**9.9**
**ROELFS-F-2007**			**10.4**							
**QUAIU-1**	**12.8**									**10.3**
**VOROBY**		**13.0**		**7.7**		**8.6**				
**NAVOJOA-M-2007**										

The data can be obtained from website http://orderseed.cimmyt.org/iwin/iwin-results-1.php.
ESWYT – Elite Spring Wheat Yield Trial: IBWSN – International Spring Bread Wheat Screening Nursery
SAWSN – Semi-arid Wheat Screening Nursery. MUNAL-1 has been released in India as SUPER-172

Table S4a. Soil-nitrate levels and soil-ammonium levels in core-soil samples taken (at 20 cm depth) near the plants of seedbeds in field-plots of BNI-isogenic lines, MUNAL-control and BNI-MUNAL (**Field Study-6a**). Three-way ANOVA using GLM with SYSTAT 14.0 (*Values are means* $\pm$ *SE of four replications)*

**Nitrogen application (kg N ha^{-1})**	**Soil nitrate (mg NO_3^- kg^{-1} soil) 1st sampling**			
	Wheat genetic stocks			
	**MUNAL-control**		**BNI-MUNAL**	
	Mean	SE	Mean	SE
**0**	1.75	*0.25*	2.00	*0.41*
**100**	33.0	*1.91*	26.3	*3.0*
**250**	68.3	*4.9*	47.4	*6.4*
**Mean**	**34.3**		**25.2**	
SE of LS Mean (Genetic stock) 2.41 (*P<0.05*)<br>SE of LS Mean (N-Tr) = 2.96 (*P<0.001*)				
**Nitrogen application (kg N ha^{-1})**	**Soil ammonium (mg NH_4^+ kg^{-1} soil)** 1st sampling			
	Wheat genetic stocks			
	**MUNAL-control**		**BNI-MUNAL**	
	Mean	*SE*	Mean	*SE*
**0**	1.50	*0.29*	3.75	*1.03*
**100**	16.00	*3.1*	45.5	*5.4*
**250**	64.3	*6.1*	105.3	*12.8*
**Mean**	**27.3**		**51.5**	
SE of LS Mean (Genetic stock) 4.3 (*P<0.001*)<br>SE of LS Mean (N-Tr) = 5.2 (*P<0.001*)				
**Nitrogen application (kg N ha^{-1})**	**Soil nitrate (mg NO_3^- kg^{-1} soil) 2nd Sampling**			
	Wheat genetic stocks			
	**MUNAL-control**		**BNI-MUNAL**	
	Mean	*SE*	Mean	*SE*
**0**	3.5	0.5	5.0	0.0
**100**	66.0	6.5	59.3	3.5
**250**	185.8	11.8	144.5	14.9
**Mean**	**85.2**		**69.6**	
SE of LS Mean (Genetic stock) 5.1 (*P<0.005*)<br>SE of LS Mean (N-Tr) = 6.3 (*P<0.001*)				
**Nitrogen application (kg N ha^{-1})**	**Soil ammonium (mg NH_4^+ kg^{-1} soil) 2nd sampling**			
	Wheat genetic stocks			
	**MUNAL-control**		**BNI-MUNAL**	
	Mean	*SE*	Mean	*SE*
**0**	5.0	0.4	5.3	0.5
**100**	6.0	0.0	5.8	0.3
**250**	74.0	7.6	100.3	8.9
**Mean**	**28.3**		**37.1**	
SE of LS Mean (Genetic stock) 3.5 (*P<0.09*)<br>SE of LS Mean (N-Tr) = 4.2 (*P<0.001*)				

Note: 1st sampling - soil cores are taken on 7th day after 2nd split N-application; 2nd sampling – soil cores were taken on 7th day after 3rd split N-application.

Table S4b. Soil mineral nitrogen as ammonium (kg N-NH_4^+ ha^{-1}) and nitrate (kg N-NO_3^- ha^{-1}), and the abundance of AOB and AOA *amoA* gene (copies g^{-1} dry soil) in soil from BNI-isogenic lines, MUNAL-control and BNI-MUNAL **(Study-6b)**. Wheat was fertilized with 200 kg N ha^{-1} in form of ammonium sulphate. Time of sampling was 30 days after fertilization. The *t*-student test was used for the comparison of Munal-Control and BNI-Munal, and the significant differences at $p < 0.05$ are marked with different letters.

	**Soil NH_4^+ content (kg NH_4^+-N ha^{-1})**	**Soil NO_3^- content (kg NO_3^--N ha^{-1})**	**AOB *amoA* genes (copies g^{-1} dry soil)**	**AOA *amoA* genes (copies g^{-1} dry soil)**
**MUNAL-Control**	0.31 ± 0.16 b	33.78 ± 1.84 a	6.04E+06 ± 9.91E+05 a	3.36E+07 ± 2.44E+06 a
**BNI-MUNAL**	1.31 ± 0.29 a	9.63 ± 2.34 b	3.31E+06 ± 5.58E+05 b	3.32E+07 ± 3.20E+06 a

Table S5. Leaf nitrate levels in MUNAL-control vs. BNI-MUNAL (BNI-isogenic lines) in field-grown plants (Field Study-6a). Three-way ANOVA using GLM with SYSTAT 14.0
(*Values are means* + *SE from four replications)*

**Nitrogen application ($kg\ N\ ha^{-1}$)**	**Leaf nitrate levels ($mg\ NO_3^-\ kg^{-1}$ FW) 1st sampling**			
	Wheat genetic stocks			
	**MUNAL-control**		**BNI-MUNAL**	
	Mean	SE	Mean	SE
**0**	28.3	*1.78*	20.2	*3.95*
**100**	42.9	*0.78*	34.1	*1.34*
**250**	57.1	*4.4*	37.5	*2.7*
**Mean**	**42.7**		**30.6**	
SE of LS Mean (Genetic stock) 1.8 (*P<0.001*)<br>SE of LS Mean (N-Tr) = 2.2 (*P<0.001*)				
**Nitrogen application ($kg\ N\ ha^{-1}$)**	**Leaf nitrate levels ($mg\ NO_3^-\ kg^{-1}$ FW)** 2nd sampling			
	Wheat genetic stocks			
	**MUNAL-control**		**BNI-MUNAL**	
	Mean	*SE*	Mean	*SE*
**0**	11.2	*1.34*	11.5	*2.2*
**100**	25.2	*0.7*	18.2	*2.4*
**250**	53.8	*3.1*	27.9	*2.2*
**Mean**	**30.0**		**19.2**	
SE of LS Mean (Genetic stock) 2.2 (*P<0.005*)<br>SE of LS Mean (N-Tr) = 2.7 (*P<0.001*)				

Note: Leaf samples are taken on 7th day after application of 2nd split nitrogen fertilizer to field plots - 1st sampling; 7th day after application of 3rd split N-fertilizer -2nd sampling; Leaf samples are collected from 10 plants from each field plot and stored at -20C before used for leaf nitrate determination. Fresh leaf tissue samples are used for NRA and GS activities determinations.

Table S6. Nitrate reductase activity (NRA) and Glutamine Synthetase Activity (GSA) in leaves of MUNAL-control *vs.* BNI-MUNAL (BNI-isogenic lines) of field-grown plants (Field Study-6a).
Three-way ANOVA using GLM with SYSTAT 14.0; (*Values are means ± SE from four replications)*

**Nitrogen application (kg N ha^{-1})**	**NRA in leaves (μmol NO_2^- g^{-1} protein h^{-1})**			
	Wheat genetic stocks			
	**MUNAL-control**		**BNI-MUNAL**	
	Mean	SE	Mean	SE
**0**	9.36	*0.42*	7.7	*0.17*
**100**	8.82	*0.39*	7.05	*0.17*
**250**	6.06	*0.17*	5.42	*0.11*
**Mean**	**8.08**		**6.72**	
SE of LS Mean (Genetic stock) 0.171 (*P<0.001*)<br>SE of LS Mean (N-Tr) = 0.21 (*P<0.001*)				
**Nitrogen application (kg N ha^{-1})**	**GSA (μmol g^{-1} protein h^{-1})**			
	Wheat genetic stocks			
	**MUNAL-control**		**BNI-MUNAL**	
	Mean	*SE*	Mean	*SE*
**0**	8.06	*0.12*	9.49	*0.21*
**100**	6.67	*0.41*	7.7	*0.36*
**250**	6.33	*0.11*	7.22	*0.3*
**Mean**	**7.02**		**8.14**	
SE of LS Mean (Genetic stock) 0.17 (*P<0.001*)<br>SE of LS Mean (N-Tr) = 0.20 (*P<0.001*)				

Note: Leaf samples were taken on 7th day after application of 2nd split nitrogen fertilizer to field plots (i.e. 1st sampling). Leaf samples are collected from 10 plants from each field plot and Fresh leaf tissue samples are used for NRA and GS activities determinations.

Table. S7. Total dry matter production, grain yields, Harvest index, tiller number, 100-seed weight in MUNAL-control vs. BNI-MUNAL (BNI-isogenic lines) at harvest under various N-treatments (Field Study-6a). Three-way ANOVA using GLM with SYSTAT 14.0; (*Values are means* + *SE from four replications)*

**Nitrogen application (kg N ha^{-1})**	**Total dry matter production (including grain wt.) at harvest (t ha^{-1})**			
	Wheat genetic stocks			
	**MUNAL-control**		**BNI-MUNAL**	
	Mean	SE	Mean	SE
**0**	12.1	*0.3*	17.6	*0.48*
**100**	17.5	*0.41*	19.1	*0.48*
**250**	19.6	*0.63*	21.2	*0.45*
**Mean**	**16.4**		**19.3**	
SE of LS Mean (Genetic stock) 0.40 (*P<0.001*)<br>SE of LS Mean (N-Tr) = 0.49 (*P<0.001*)				
**Nitrogen application (kg N ha^{-1})**	**Grain Yield (t ha^{1})**			
	Wheat genetic stocks			
	**MUNAL-control**		**BNI-MUNAL**	
	Mean	*SE*	Mean	*SE*
**0**	4.5	*0.13*	6.8	*0.22*
**100**	7.0	*0.28*	7.7	*0.16*
**250**	7.6	*0.34*	8.8	*0.3*
**Mean**	**6.4**		**7.8**	
SE of LS Mean (Genetic stock) 0.164 (*P<0.001*)<br>SE of LS Mean (N-Tr) = 0.201 (*P<0.001*)				
**Nitrogen application (kg N ha^{-1})**	**Harvest Index HI (%)**			
	Wheat genetic stocks			
	**MUNAL-control**		**BNI-MUNAL**	
	Mean	*SE*	Mean	*SE*
**0**	37.1	*0.47*	38.6	*0.4*
**100**	40.1	*0.87*	40.5	*0.21*
**250**	38.6	*0.83*	41.4	*0.57*
**Mean**	**38.6**		**40.2**	
SE of LS Mean (Genetic stock) 0.32 (*P<0.003*)<br>SE of LS Mean (N-Tr) = 0.39 (*P<0.001*)				
**Nitrogen application (kg N ha^{-1})**	**No. of tillers per plant**			
	Wheat genetic stocks			
	**MUNAL-control**		**BNI-MUNAL**	
	Mean	*SE*	Mean	*SE*
**0**	9.9	*0.39*	12.9	*0.38*
**100**	13.0	*0.21*	14.1	*0.67*
**250**	16.2	*0.61*	16.9	*0.49*
**Mean**	**13.0**		**14.6**	
SE of LS Mean (Genetic stock) 0.32 (*P<0.002*)<br>SE of LS Mean (N-Tr) = 0.39 (*P<0.001*)				

Nitrogen application (kg N ha^{-1})	100 seed weight (g)			
	Wheat genetic stocks			
	**MUNAL-control**		**BNI-MUNAL**	
	Mean	*SE*	Mean	*SE*
**0**	4.9	*0.02*	5.3	*0.06*
**100**	4.8	*0.01*	5.0	*0.05*
**250**	4.6	*0.07*	4.8	*0.09*
**Mean**	**4.8**		**5.0**	
SE of LS Mean (Genetic stock) 0.037 (*P<0.001*)<br>SE of LS Mean (N-Tr) = 0.046 (*P<0.001*)				

Table S8a. Seed protein quality of BNI-isogenic lines of ROELF-control, BNI-ROELF, MUNAL-control, BNI-MUNAL grown in two field sites in Mexico (Obregon and Elbatan) during 2019 and 2020

**Field study**	**Genetic stock**	**GPRO (%)**	**FPRO (%)**	**SDSS (Ml)**	**LV (mL)**	**BCS (-)**	**HMW-GS composition**			**LMW-GS composition**		
							Glu-A1	*Glu-B1*	*Glu-D1*	*Glu-A3*	*Glu-B3*	*Glu-D3*
ElBatan2019	ROELF-Control	13.6	11.9	20.0	835	Very Good	*b*	*i*	*d*	b	h	b
	BNI-ROELFS (BC_5)	14.7	12.9	17.5	845	Very Good	*b*	*a*	*d*	c	h	b
Obregon 2020	ROELF-Control	13.0	11.2	16.0	765	Good	*b*	*i*	*d*	b	h	b
	BNI-ROELFS (BC_5)	13.8	12.0	15.0	725	Fair	*b*	*i*	*d*	c	h	b
	BNI-ROELFS (BC_7)	13.2	11.2	16.0	745	Good	*b*	*i*	*d*	b	h	b
ElBatan2019	MUNAL-control	14.8	12.8	18.0	840	Very Good	*b*	*a*	*d*	c	h	b
	BNI-MUNAL (BC_6)	14.1	12.2	18.5	800	Good	*b*	*c*	*d*	c	h	b
ElBatan2020	MUNAL-control	13.8	11.9	13.5	790	Very Good	*b*	*a*	*d*	c	b	b
	BNI-MUNAL (BC_6)	12.5	10.9	12.0	685	Fair	*b*	*c*	*d*	c	h	e

GPRO: grain protein content; FPRO: flour protein content; **SDSS**: SDS-sedimentation volume; **LV**: bread loaf volume; **BCS**: bread crumb structure; **HMW-GS**: high molecular weight glutenin alleles; **LMW-GS**: low molecular weight glutenin alleles.

Note: Nitrogen fertilizer application is 250 kg N ha^{-1} in all the above field trials in Mexico

Table S8b. Seed protein levels of BNI-isogenic lines of MUNAL-control and BNI-MUNAL grown in JIRCAS field study in Japan (2019-2020) (Study 6a).

Field study (2019-2020)	Genetic stock	Nitrogen application (kg N ha^{-1})	Grain protein (%)
JIRCAS field study, Tsukuba, Japan			
	MUNAL-control	0 kg N ha^{-1}	12.55
	BNI-MUNAL	0 kg N ha^{-1}	12.18
	MUNAL-control	250 kg N ha^{-1}	13.51
	BNI-MUNAL	250 kg N ha^{-1}	12.74

Table S9. Grain yield and days to maturity of BNI-MUNAL and BNI-ROELFS along with controls in two field sites of Obregon CIMMYT experimental station in Mexico during 2019 and 2020 season under high-N input (250 kg N ha^{-1}) systems.

Wheat genetic stock	Maturity (days)					Grain Yield (t ha^{-1})			
	2019		2020			2019		2020	
	Obregon 1	Obregon 2	Obregon 1	Obregon 2		Obregon 1	Obregon 2	Obregon 1	Obregon 2
MUNAL-control	125	133	125	121		7.6	5.5	7.2	6.8
BNI-MUNAL (BC_6)	129^{*}	133	126^{ns}	121^{ns}		7.9^{ns}	7.7^{*}	7.9^{ns}	6.6^{ns}
ROELFS-control	126	131	123	119		7.3	6.7	7.5	7.0
BNI-ROELFS (BC_5)	127^{ns}	134^{*}	126^{*}	122^{*}		6.9^{ns}	5.4^{*}	6.3^{*}	5.7^{*}

Note: The experimental design of the individual experiments was a randomized complete block and the combined ANOVA was done across years and sites. The statistical software used was SAS, Copyright © 2016 by SAS Institute Inc., Cary, NC, USA, SAS ® Proprietary Softward 9.4 (TS1M5) and the procedure used was a general linear model, GLM.
